# Self-Assembly of Toll-Like Receptor (TLR2/6) Agonist Lipidated Amino Acid or Peptide Conjugates: Distinct Morphologies and Bioactivities

**DOI:** 10.1021/acs.bioconjchem.5c00051

**Published:** 2025-04-02

**Authors:** Valeria Castelletto, Lucas R. de Mello, Juliane Pelin, Ian W Hamley

**Affiliations:** † School of Chemistry, Food Biosciences and Pharmacy, University of Reading, Whiteknights, Reading RG6 6AD, U.K.; ‡ Currently at Departamento de Ciências Farmacêuticas, Universidade Federal de São Paulo, Diadema, São Paulo 09913-030, Brazil

## Abstract

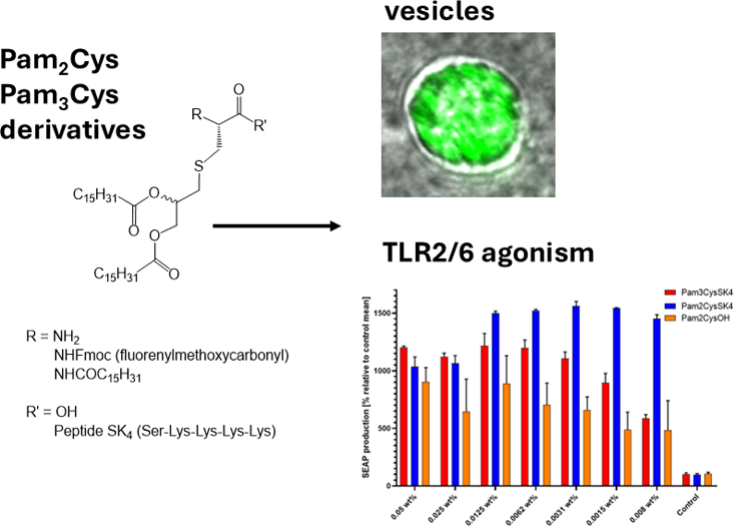

Toll-like receptor (TLR) agonists are of interest in immunotherapy and cancer vaccines. The most common agonists of TLR2 are based on Pam_2_Cys or Pam_3_Cys. In the former, two palmitoyl (Pam) fatty acids are linked to a glycerylcysteine motif by ester linkages. Pam_3_Cys is analogous but contains an extra Pam group on the α-amine. Here, we compare the self-assembly in aqueous solution of the parent Pam_2_CysOH and Pam_3_Cys amino acid conjugates to that of Pam_2_CysSK_4_ and Pam_3_CysSK_4_ which are potent TLR2 agonists bearing the CysSK_4_ peptide sequence. All four conjugates exhibit a critical aggregation concentration above which self-assembled structures are formed. We find through a combination of small-angle X-ray scattering (SAXS), cryogenic transmission electron microscopy (cryo-TEM), and confocal fluorescence microscopy remarkable differences in self-assembled nanostructures. Pam_2_CysOH and Pam_3_CysOH both form unilamellar vesicles, although these are larger for the latter compound, an effect ascribed to enhanced membrane rigidity. This is in contrast to previously reported morphologies for Pam_2_CysSK_4_ and Pam_3_CysSK4, which are spherical micelles or predominantly wormlike micelles, respectively [Hamley, I. W.; et al. *Toll-like Receptor Agonist Lipopeptides Self-Assemble into Distinct Nanostructures*. Chem. Comm. 2014, 50, 15948-15951]. We also examine the effect of introduction in the bulky *N*-terminal Fmoc [fluorenylmethoxycarbonyl] group on the self-assembly of Fmoc-Pam_2_CysOH. This compound also forms vesicles (above a critical aggregation concentration, determined from dye probe fluorescence experiments) in aqueous solution, larger than those for Pam_2_CysOH and with a population of perforated/compound vesicles. The carboxyl-coated (and amino-coated for Pam_2_CysOH) vesicles demonstrated here represent a promising system for future development toward bionanotechnology applications such as immune therapies. Conjugates Pam_2_CysOH, Pam_2_CysSK_4_, and Pam_3_CysSK_4_ show good cytocompatibility at low concentrations, and in fact, the cell compatibility extends over a wider concentration range for Pam_2_CysOH. The TLR2/6 agonist activity was assessed using an assay that probes secreted alkaline phosphatase (SEAP) in NF-κB-SEAP reporter HEK293 cells expressing human TLR2 and TLR6, and Pam_2_CySOH shows significant activity, although not to the extent of Pam_2_CysSK4 or Pam_3_CysSK_4_. Thus, Pam_2_CysOH in particular is of interest as a vesicle-forming TLR2/6 agonist and stimulator of immune response.

## Introduction

Lipopeptides are emerging as a class of biobased or bioderived molecules with a remarkable range of applications, among which a particular current highlight is the antidiabetic/weight control gut hormone peptide-based compounds semaglutide and tirzepatide, which also show promising potential for many other important healthcare applications. Lipopeptides (one type of peptide amphiphile, PA) also have demonstrated roles as materials for biomedicine and tissue engineering, as antimicrobials, in biocatalysis, and in many other areas.^1−10^ Lipopeptides can self-assemble into different nanostructures depending on their structure (lipid chain type, length, and peptide sequence) as well as the solution conditions.^2−4,7,11−15^ A diversity of nanostructures has been observed including nanofibrils, nanosheets, nanotubes, vesicles, and micelles. The most commonly reported are fibrillar structures, which are stabilized by β-sheet intermolecular hydrogen bonding. Lipopeptide micelles have been observed as a result of the self-assembly of various types of peptides, especially those with short or disordered sequences where β-sheet aggregation is suppressed. Vesicular structures are infrequently observed for lipopeptide (or indeed peptide) systems since there is a particular constraint on bilayer packing of molecules in the membrane walls with intrinsic curvature for vesicle formation. Examples of vesicle-forming peptide systems include glycine-rich surfactant-like peptides,^16^ sequenced peptides,^17^ Boc-diphenylalanine [Boc: *tert*-butoxycarbonyl] in organic solvents,^18^ proline-based surfactant-like peptides,^19^ or peptide bolaamphiphiles.^20^

The innate immune response depends on the recognition of pathogens. Toll-like receptors (TLRs) are cell membrane proteins (with some TLR types located in intracellular vesicles) that serve as pattern recognition receptors (PRRs) that can recognize pathogen-related molecules (that can be distinguished from host molecules), which are known as pathogen-associated molecular patterns (PAMPs). Lipopeptides have been used for the development of vaccines, their adjuvants, and agents for cancer immunotherapy,^9^ and one of the most active sequences is the lipid-linked toll-like receptor (TLR) agonist hexapeptide CSK_4_ (Cys-Ser-Lys-Lys-Lys-Lys).^9,21^ The lipid glyceryl-cysteine component is derived from bacterial lipopeptides (which stimulate a strong immune response).^22^ The CSK_4_ sequence shows activity as an adjuvant^23^ and is derived from the Pam_3_CSSNAK [Pam: palmitoyl, C_16_] N-terminal domain of the murein (peptidoglycan) lipoprotein in the outer cell membrane of *E. coli*.^24,25^ Due to their strong adjuvant activity, Pam_2_Cys and Pam_3_Cys-based conjugates have been extensively examined. Further details on TLR lipopeptides, including examples and applications, are discussed elsewhere.^9,21,26,27^ There have been fewer studies on the self-assembly of this class of molecules. We examined the conformation and self-assembly of PamCSK_4_, Pam_2_CSK_4_, and Pam_3_CSK_4_.^28^ The former two molecules form spherical micelles with the peptide in a disordered conformation, whereas the latter forms flexible wormlike micelles (coexisting with globular structures) with a β-sheet secondary structure and bilayer molecular packing. These structures were later confirmed by molecular dynamics simulations.^29^

We were motivated to examine the self-assembly and bioactivity of minimal Pam_2_Cys- and Pam_3_Cys-based molecules, specifically to investigate whether these thioglycerol “scaffolds” bearing one or two palmitoyl chains, but without peptide sequences as in CSK_4_ or CSSNAK, are able to self-assemble and whether they show any bioactivity. Here, we compare the self-assembly in aqueous solution of the amino acid-based conjugates Pam_2_CysOH, Pam_3_CysOH, and Fmoc-Pam_2_Cys and the TLR-agonist peptide conjugates Pam_2_CysSK_4_ and Pam_3_CysSK_4_. To the best of our knowledge, Pam_2_CysOH, Pam_3_CysOH, and Fmoc-Pam_2_Cys have not previously been studied. We show that in fact, they do form self-assembled nanostructures and act as TLR2/6 agonists. The critical aggregation concentration (CAC) is determined from lipophilic dye probe fluorescence assays. Confocal microscopy is used to image vesicle structures along with cryo-TEM which also elucidates the (smaller scale) micellar structures formed by the peptide conjugates. SAXS provides unique detailed information about the size and shape of nanostructures. It shows that the vesicle structures formed by Pam_2_CysOH and Pam_3_CysOH are unilamellar and confirms the distinct self-assembly behavior of Pam_2_CysSK_4_ in spherical micelles and Pam_3_CysSK_4_ in wormlike micelles. Vesicle size distributions are determined by dynamic light scattering, and peptide conformations are probed using circular dichroism spectroscopy. In addition, we examined the cytocompatibility of the conjugates using MTT assays. We also measure the TLR2/6 agonist activity, comparing the new Pam_2_CysOH conjugate with the well-known Pam_n_CSK_4_ lipopeptide analogues using an assay that probes secreted embryonic alkaline phosphatase (SEAP) by NF-κB-SEAP reporter-engineered HEK293 cells expressing human TLR2 and TLR6.

## Methods

### Materials and Sample Preparation

**Scheme 1:**
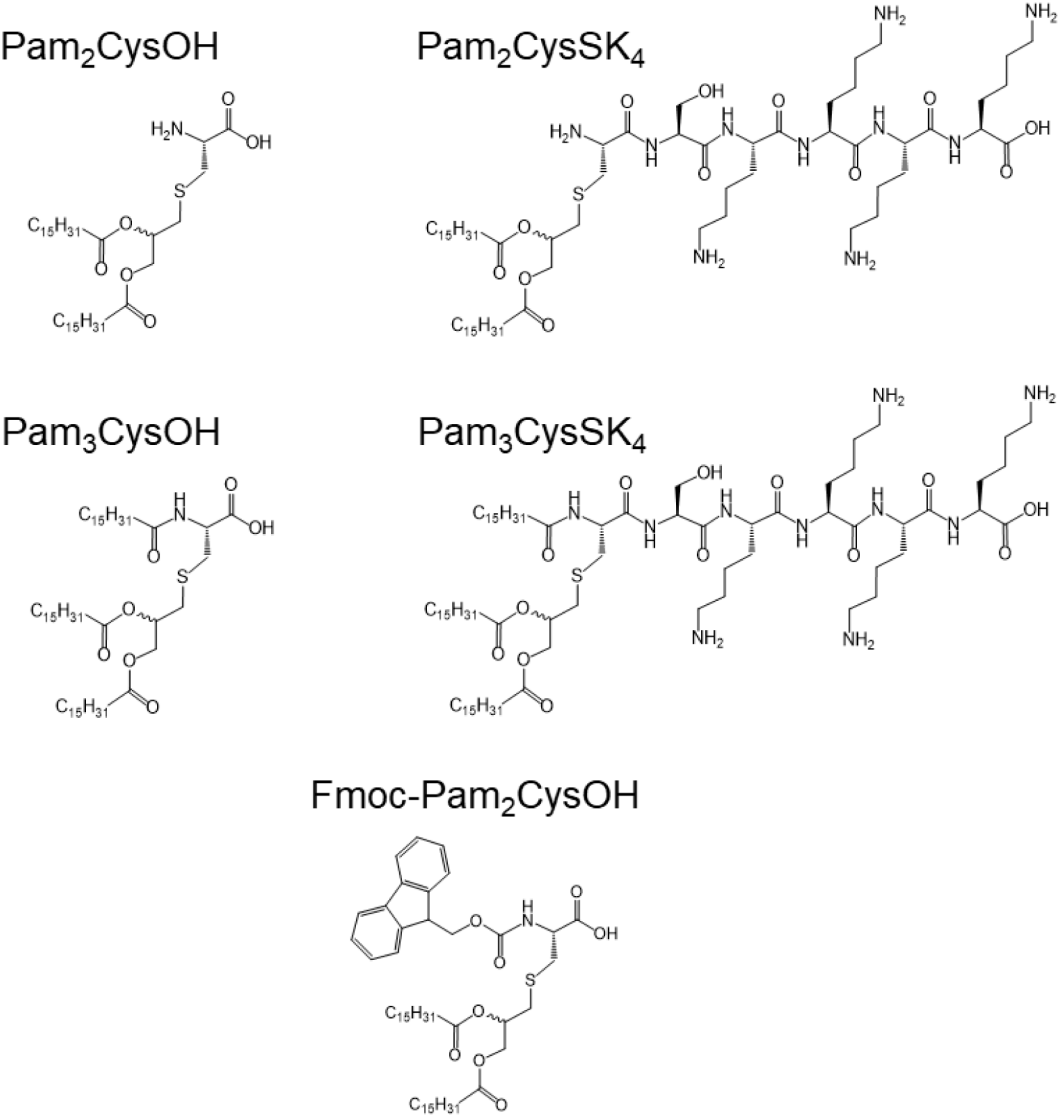
Molecular Structures

Lipopeptides Pam_2_CysOH, Pam_3_CysOH, Pam_2_CysSK_4_, and Pam_3_CysSK_4_ (Scheme 1) were custom-synthesized by Peptide Synthetics (Peptide Protein Research Ltd., Farnham, UK) and supplied as TFA salts. The molar mass measured by ESI-MS for Pam_2_CysOH is 672.05 g mol^–1^ (671.52 g mol^–1^ expected), and for Pam_3_CysOH, the measured molar mass is 910.48 g mol^–1^ (909.75 g mol^–1^ expected). The molar mass measured by ESI-MS for Pam_2_CysSK_4_ is 1271.8 g mol^–1^ (1270.9 g mol^–1^ expected), and for Pam_3_CysSK_4_, the measured molar mass is 1510.2 g mol^–1^ (1509.1 g mol^–1^ expected). Fmoc-Pam_2_CysOH was also prepared by Peptide Synthetics and supplied as a TFA salt. The molar mass measured by ESI-MS for Fmoc-Pam_2_CysOH is 894.3 g mol^–1^ (894.3 g mol^–1^ expected). The purity by HPLC (0.1% TFA in acetonitrile/water gradient) is >95% for all samples.

Solutions were prepared using the thin-layer hydration method, previously used to prepare liposomes.^30^ A peptide film was produced by weighing a quantity of peptide, dissolving it in chloroform, and drying it under a stream of nitrogen. The peptide film was then placed in a vacuum chamber for 2 h to remove traces of organic solvent. A peptide solution was obtained by resuspending the peptide film in water, repeatedly vortexing at 1800 rpm, and heating at 65 °C (above the melting temperature of palmitic acid, 62.9 °C) for 5 min. The peptide solution was then left to equilibrate before the experiments. The final peptide concentrations were calculated in weight percent= (100 × weight of peptide) /(weight of peptide + weight of water). For example, 1 mg of peptide powder was dissolved in chloroform to make a peptide film. The peptide was resuspended in 99 mg of solvent to provide a final concentration of 1 wt % peptide.

The pH values of 0.5 wt % Pam_2_CysOH, 0.5 wt % Pam_2_CysSK_4_, 0.5 wt % Pam_3_CysOH, 0.5 wt % Pam_3_CysSK_4_, and 0.1 wt % Fmoc-Pam_2_CysOH in water were 3.6, 2.6, 4.8, 2.8, and 7.8, respectively. The pH of the acidic solutions was adjusted to pH 7 by NaOH titration.

A separate set of samples was prepared for confocal microscopy experiments by following the procedure described in the paragraph above. A solution containing 3 × 10^–5^ wt % Nile red or 3 × 10^–4^ wt % Rhodamine B was used instead of water as a solvent to stain the peptides.

### Critical Aggregation Concentration (CAC) Fluorescence Assay

The CAC was determined by fluorescence assays using Nile red. For this, the samples were incubated in a solution of water containing 5 μM Nile red. The excitation wavelength was set at 550 nm, and the bandwidths were set at 5 nm. The fluorescence was recorded using a Varian Cary Eclipse Spectrofluorometer (Agilent, USA).

### Circular Dichroism (CD) Spectroscopy

Far-UV CD spectra were collected using a Chirascan spectropolarimeter (Applied Photophysics, Leatherhead, UK) equipped with a thermal controller. Spectra were recorded from 180 to 400 nm. Samples were mounted in a quartz cell with detachable windows, with a 0.01 mm path length, or in a quartz bottle with a 1 mm path length. The CD signal from the samples was corrected by subtracting the water background. The CD spectra were smoothed using Chirascan software for data analysis. The residual of the calculation was chosen to oscillate around the average to avoid artifacts in the smoothed curve. CD data, measured in mdeg, were normalized to molar ellipticity using the molar concentration of the sample and the cell path length. The absorbance spectra were measured by the instrument simultaneously with the CD and are presented for 0.1 wt % Fmoc-Pam_2_CysOH in Supporting Information.

### Dynamic Light Scattering

The hydrodynamic radius of the vesicles, *R*_H_, was calculated from the experimental DLS data. DLS data were measured using a Zetasizer Nano ZS from Malvern Instruments or an ALV/CGS-3 Compact Goniometer System with an ALV/LSE-5003 correlator. For the Malvern instrument, an aliquot of 120 μL of sample was placed inside a quartz cell with a 1 mm path length. The DLS was measured at a fixed scattering angle of 175°. The *R*_H_ was calculated from cumulant fitting of the DLS autocorrelation functions using Malvern software. For the measurements using the ALV/CGS-3 Compact Goniometer System, 1 mL of solution was loaded into a glass tube with a 1 cm internal diameter. The system uses vertically polarized incident light with a wavelength of 632.8 nm. Measurements were performed at an angle θ = 90 ° to the incident beam. The intensity autocorrelation functions were analyzed by the constrained regularized CONTIN method^31^ to obtain size distributions of *R*_H_.

### Laser Scanning Confocal Microscopy

Imaging was performed using a Nikon A1 HD25/A1R HD25 confocal microscope. Solutions were prepared as detailed in the sample preparation method. A drop of the sample was placed on a microscope slide, and a microscope coverslip was placed on the drop. The edges of the microscope coverslip were sealed with varnish to avoid sample evaporation, and the sample was allowed to rest for approximately 20 min before examination. Experiments were performed using a Plan Apo λ 100× oil lens or a Plan Apo VC 20× DIC N2 lens. Pinhole sizes were 25.25 μm, 20.43 μm, 35.76 μm, or 24.27 μm. Solutions stained with Nile red were excited at 561 nm, and the emission was measured at 595 nm. Transmission detector (TD) images in bright-field transmission mode were generated by illuminating the sample with 405 nm light. For imaging with Rhodamine B, 500 μL of a 0.03 wt % aqueous solution of the dye was added to 500 μL of a 0.049 wt % Fmoc-Pam_2_CysOH aqueous solution. Fluorescence images were obtained from a drop of the sample deposited on a glass slide, with an excitation wavelength of 561 nm. The bandpass filters on the confocal microscope were set for a window between 565 and 590 nm, and the objective lenses selected for this assay provided magnifications of 20×, 60×, and 100×.

### Solution Small-Angle X-ray Scattering (SAXS)

SAXS experiments were performed on beamline B21^32^ at Diamond (Didcot, UK). The sample solutions were loaded into a 96-well plate of an EMBL BioSAXS robot and then injected via an automated sample exchanger into a quartz capillary (1.8 mm internal diameter) within the X-ray beam. The quartz capillary was enclosed in a vacuum chamber to avoid parasitic scattering. After the sample was injected into the capillary and reached the X-ray beam, the flow was stopped during SAXS data acquisition. Beamline B21 operates with a fixed camera length (3.9 m) and fixed energy (12.4 keV). The images were captured by using a PILATUS 2 M detector. Data processing was performed by using dedicated beamline software, ScÅtter.

### Cryogenic Transmission Electron Microscopy (Cryo-TEM)

Samples were deposited onto Quantifoil R2/1 holey carbon Cu/Rh grids with a hole size of 2 μm and a 1 μm spacing. Prior to use, the grids were plasma-cleaned using a Quorum SC7620 glow discharge system for 1 min at an atmospheric pressure of 0.1 mbar and a current of 30 mA. A FEI Vitrobot Mark IV plunge freezing system was used for vitrification, with the climate chamber kept at 100% humidity and maintained at 4 °C. For each grid, 3.5 μL of sample was applied and blotted twice with a blot force of 3 and a blot time of 3.5 s before plunging into liquid ethane at a temperature of −180 °C. The vitrified grids were then clipped using the Thermo-FEI clipping station and stored in a liquid nitrogen storage system. Images from the vitrified samples were acquired using a Thermo-FEI Glacios field emission microscope operating at 200 kV coupled with a Falcon4i direct electron detector and Selectris energy filter. EPU software was used to select targets and acquire images at specified magnifications. Images were collected in bright-field mode with parallel electron beam illumination onto the specimen and zero-loss energy filtering with a slit width of 5 eV.

### Cytocompatibility

Assays of mitochondrial activity using MTT [3-(4,5-dimethylthiazol-2-yl)-2,5-diphenyltetrazolium bromide] were conducted to determine cytocompatibility. Initially, HEK 293T cells (ATCC) were kindly donated by Prof. Mark Dallas (University of Reading) and cultivated in T75 flasks at 37 °C under a 5% CO_2_ atmosphere using DMEM medium supplemented with 10% fetal bovine serum plus antibiotics. After expansion, the cells were seeded into 96-well plates at a confluence of 2 × 10^4^ cells/well with supplemented DMEM. Then the wells were washed 3× with PBS and incubated for 72 h in media containing different concentrations of the Pam conjugates (dissolved in DMEM with sonication). After incubation, the cells were washed again 3× with PBS, and 100 μL of DMEM without phenol red +5 μg/mL of MTT was added to each well. The plate was incubated for 4 h inside an incubator at 37 °C, protected from light. After 4 h incubation, the DMEM was removed, and 100 μL of DMSO was added to each well to solubilize the resulting formazan crystals, followed by incubation for 30 min at 37 °C, protected from light. The absorbance was measured at 560 nm using an Infinite F50 microplate reader (TECAN, Switzerland) and the software Magellan. Resulting values were statistically analyzed using ANOVA (*n* = 3) with Bonferroni correction for multiple assays

### Secreted Embryonic Alkaline Phosphatase (SEAP) Assay

Human HEK-Blue hTLR2-TLR6 cells were purchased from InvivoGen (Toulouse, France). As recommended by the manufacturer, for the first 2 passages, the cells were expanded without the use of selective antibiotics in DMEM + 10% of fetal bovine serum (without antibiotics) and incubated at 37 °C under an atmosphere of 5% CO_2_. After expansion, the cells were cultivated in the presence of HEK-Blue Selection (InvivoGen, Toulouse, France), a mix of antibiotics, for at least two passages. For the SEAP quantification assays, cells were detached with trypsin, and 2 × 10^4^ cells/well were seeded on 96-well plates and incubated at 37 °C for 24 h for recovery and attachment before the assay. Finally, the plates were washed with PBS and incubated in HEK-Blue Detection (InvivoGen, Toulouse, France) media without serum + Pam conjugates for 24 h inside a cell incubator. The controls were incubated using only HEK-Blue Detection media without serum. After this final incubation, the resulting SEAP production was quantified by measuring the absorbance at 620 nm using Tecan Infinite F50 (Tecan, Männedorf, Switzerland) with the Magellan software.

## Results

**Figure 1:**
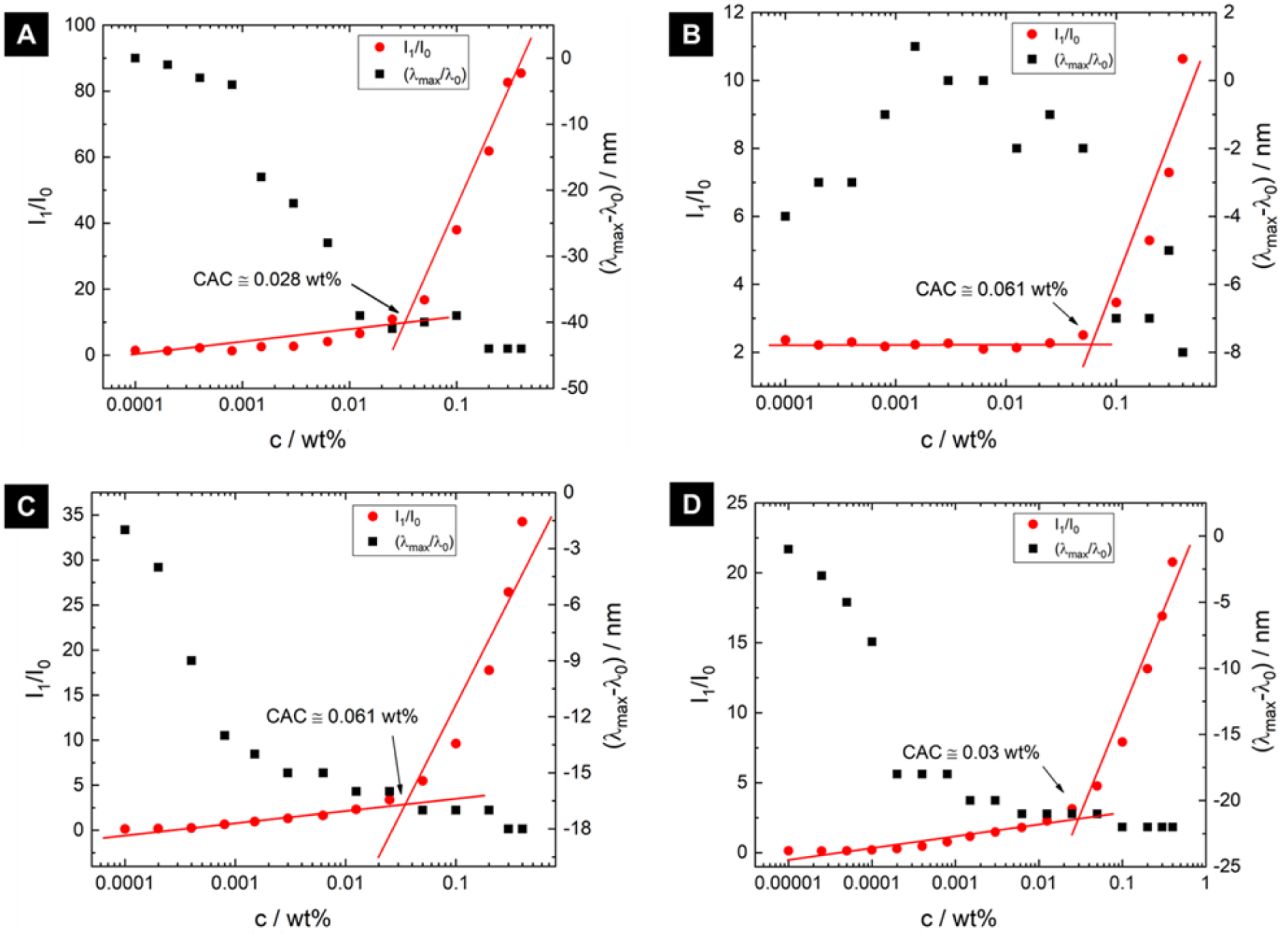
Nile red fluorescence assay data to determine critical aggregation concentration (CAC). Left axis: fluorescence intensity ratio *I*_1_/*I*_0_, where *I*_0_ is the intensity from a Nile red solution without conjugate (red dots). Right axis: blue shift represented by the wavelength shift (λ_max_ – λ_0_)/nm (black dots) for (A) Pam_2_CysOH, (B) Pam_3_CysOH, (C) Pam_2_CysSK_4_, and (D) Pam_3_CysSK_4_.

We first determined whether the conjugates Pam_2_CysOH, Pam_3_CysOH, Pam_2_CysSK_4_, and Pam_3_CysSK_4_ shown in Scheme 1 exhibit concentration-dependent aggregation, through a fluorescence assay of critical aggregation concentration (CAC) [due to the presence of the bulky N-terminal aromatic group, Fmoc-Pam_2_CysOH is considered separately below]. The assays were performed using Nile red, which is a neutral lipophilic dye,^33−35^ and the fluorescence peak intensity (normalized by that of the control Nile red solution), *I*/*I*_0_, and peak wavelength shift are plotted for each conjugate in Figure 1. The original spectra are shown in Figure S1. In Figure 1, the CAC is most evident from breakpoints in *I*/*I*_0_ although it also coincides with the concentration above which there is no longer a concentration-dependent blue shift in peak position. All four conjugates show similar CAC values in the range of 0.028–0.061 wt % with no notable trends in terms of dependence on the number of Pam chains or the amino acid/peptide sequence. All samples presented some degree of blue shift when compared to the Nile red control, this being notably greater for Pam_2_CysOH and lower for Pam_3_CysOH (which is also the case for the change in *I*/*I*_0_ values). A blue shift in Nile red fluorescence intensity arises because the dye fluorescence is influenced by the polarity of the microenvironment^33−36^ and indicates that aggregates have a polar core that influences the quantum yield of Nile red. The highly lipophilic nature of the dye leads to a significant blue shift in fluorescence peak position in the presence of self-assembled lipids.^33,36^

**Figure 2:**
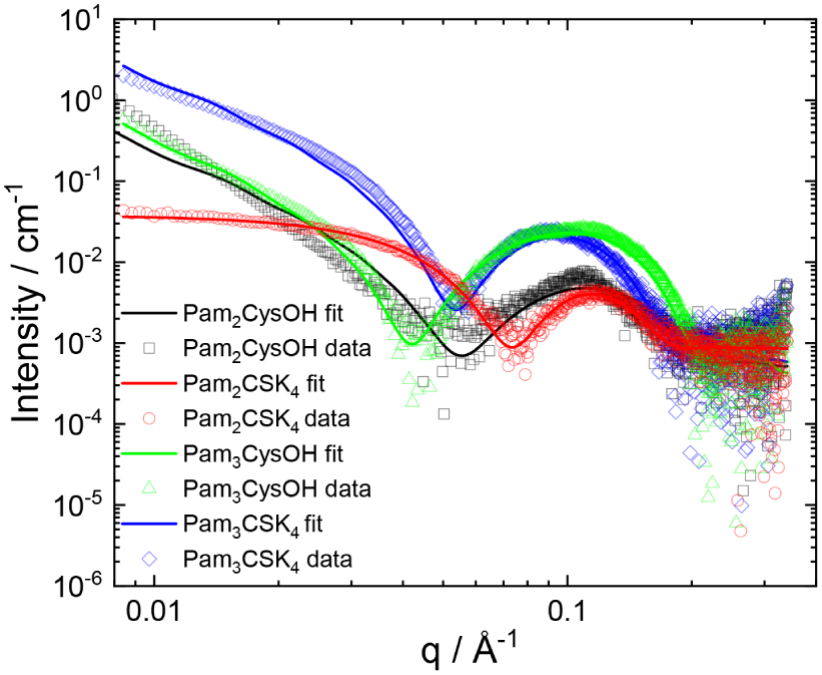
SAXS data from 0.5 wt % aqueous solutions under conditions as indicated. Open symbols are measured data, and the solid lines are fits to form factors, as described in the text (fitting parameters in Table S1). For ease of visualization, only every third measured data point is plotted.

A combination of SAXS, cryogenic-TEM, and confocal microscopy was used to probe the self-assembled structures in solution. SAXS data are shown in Figure 2 along with fitted form factors. The fit parameters are listed in Table S1. The data in Figure 2 show that Pam_2_CysSK_4_ forms core–shell spherical micelles at both pH values studied, consistent with our previous report.^28^ In contrast, the intensity profile for Pam_2_CysOH is very different from that of Pam_2_CysSK_4_, with a distinct slope at low *q* and a much broader and shifted form factor maximum. The data correspond to bilayer structures, consistent with the presence of vesicle-like structures revealed by confocal microscopy (discussed below).

We previously showed that Pam_3_CysSK_4_ self-assembles into wormlike micelles based on a bilayer arrangement of the molecules (coexisting with globular micelles).^28,29^ The SAXS data shown in Figure 2 are consistent with this, showing a form factor that can be fitted using a Gaussian bilayer model (parameters listed in Table S1) which represents an electron density profile across the bilayer described by three Gaussian functions (one for the electron-depleted lipid interior and two for the electron-rich peptide surfaces of the bilayers). In contrast, the SAXS data for Pam_3_CysOH show distinct features in that the form factor maxima are much stronger and wider. This is a signature of unilamellar vesicle structures.^37^

**Figure 3:**
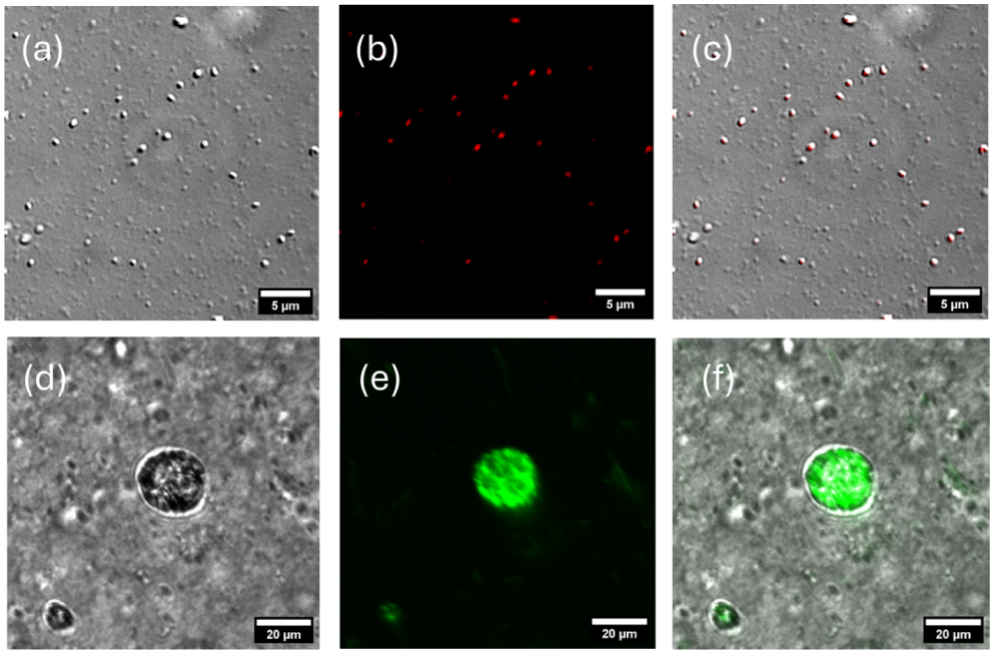
Confocal microscopy images from 0.5 wt % solutions of samples stained with 3 × 10^–5^ wt % Nile red. (a,b,c) Pam_2_CysOH and (d,e,f) Pam_3_CysOH. (a,d) Bright-field image, (b,e) fluorescence image (Nile red fluorescence channel), and (c,f) merged images.

SAXS reveals that the two conjugates lacking the SK_4_ peptide sequence, i.e., Pam_2_CysOH and Pam_3_CysOH, form unilamellar vesicles, distinct from the spherical micelle and wormlike micelle (+globule) structures for the two peptide-bearing analogues. SAXS does not provide information on the vesicle size, which is too large to be obtained considering the *q* range covered in the SAXS experiment. We thus used confocal microscopy to image nanostructures and dynamic light scattering (DLS) to obtain average vesicle sizes. Confocal microscopy images shown in Figure 3 indicate that Pam_2_CysOH forms small vesicles, 1 μm or less in diameter, whereas for Pam_3_CysOH, a population of larger vesicles up to 20 μm in size was noted. In addition, a population of fibrillar structures could be distinguished (Figure S2). The difference in size of the vesicles comparing the two molecules is confirmed by DLS data in Figure S3. The size difference indicates differences in bilayer stiffness (bending modulus) since the thickness is very similar, as shown by SAXS (Table S1). The difference in stiffness may arise from the distinct charge of the hydrophilic units for Pam_2_CysOH compared to Pam_3_CysOH and Fmoc-Pam_2_CysOH, since for Pam_2_CysOH, both amino and carboxyl groups are expected to be present, whereas for the latter two molecules, only carboxyl groups are present (Scheme 1). The bilayer rigidity may also be modulated by distinct packing of the molecules with greater lipid chain interdigitation in the case of Pam_3_CysOH.

**Figure 4:**
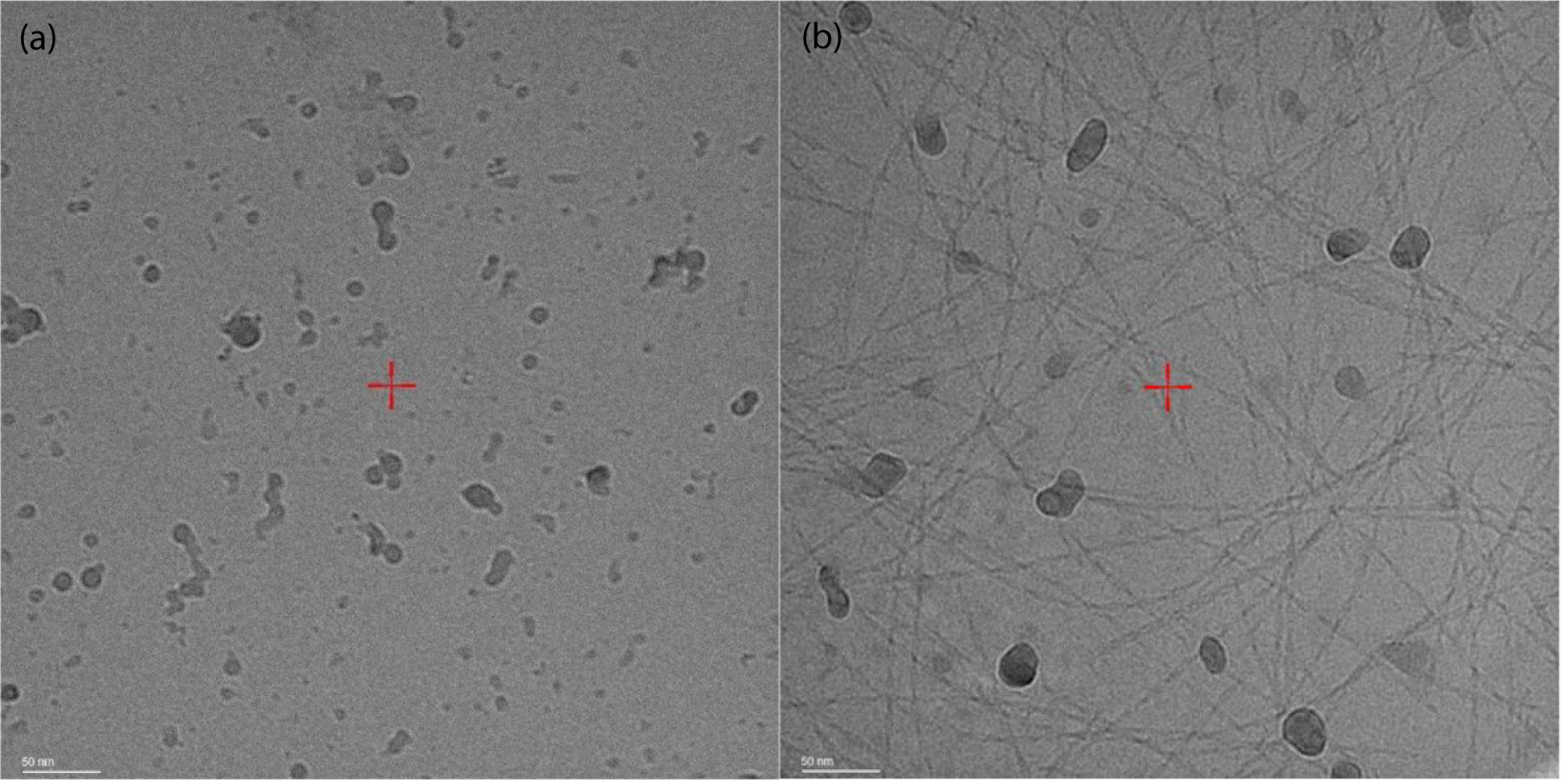
Cryo-TEM images from 0.5 wt % pH 7 solutions of (a) Pam_2_CysOH and (b) Pam_3_CysOH.

The solutions contain vesicles with a range of sizes. Structures smaller than 250 nm are usually beyond the resolution limit of confocal microscopy, and therefore, cryogenic-TEM images were also obtained to detect the population of smaller vesicles. Cryo-TEM images in Figure 4 show small vesicles for Pam_2_CysOH and larger vesicles for Pam_3_CysOH. In addition to the vesicles, the cryo-TEM image reveals a population of fibrils, consistent with the confocal microscopy (Figure S2), although a population of fibrils was not evident in SAXS data fitting. Since SAXS provides sample-averaged information on nanostructure (i.e., in the volume probed by the X-ray beam, a much larger region than imaged by confocal or cryo-TEM microscopy), this suggests that the fraction of fibrils is low.

The secondary structure of Pam_2_CysSK_4_ and Pam_3_CysSK_4_ was probed using circular dichroism (CD) spectroscopy. The spectra shown in Figure S4 are consistent with our previous report, showing a random coil structure for Pam_2_CysSK_4_ and a β-sheet structure for Pam_3_CysSK_4_.^28^ These conformations correlate to the observed micelles for Pam_2_CysSK_4_ and wormlike micelles for Pam_3_CysSK_4_^28^ (additional cryo-TEM images are provided in Figure S5).

We also investigated the solution self-assembly of Fmoc-Pam_2_CysOH (Scheme 1) that is the analogue of Pam_2_CysOH but with the Fmoc protecting group, commonly used in peptide synthesis, still N-terminally attached. The Fmoc group has been used extensively to promote self-assembly in conjugates to peptides or amino acids since it can undergo π-π stacking interactions that can drive aggregation.^38^ We examined whether it has any influence on self-assembly in this class of conjugates and also used it as a reporter group in fluorescence assays of critical aggregation concentration.

**Figure 5:**
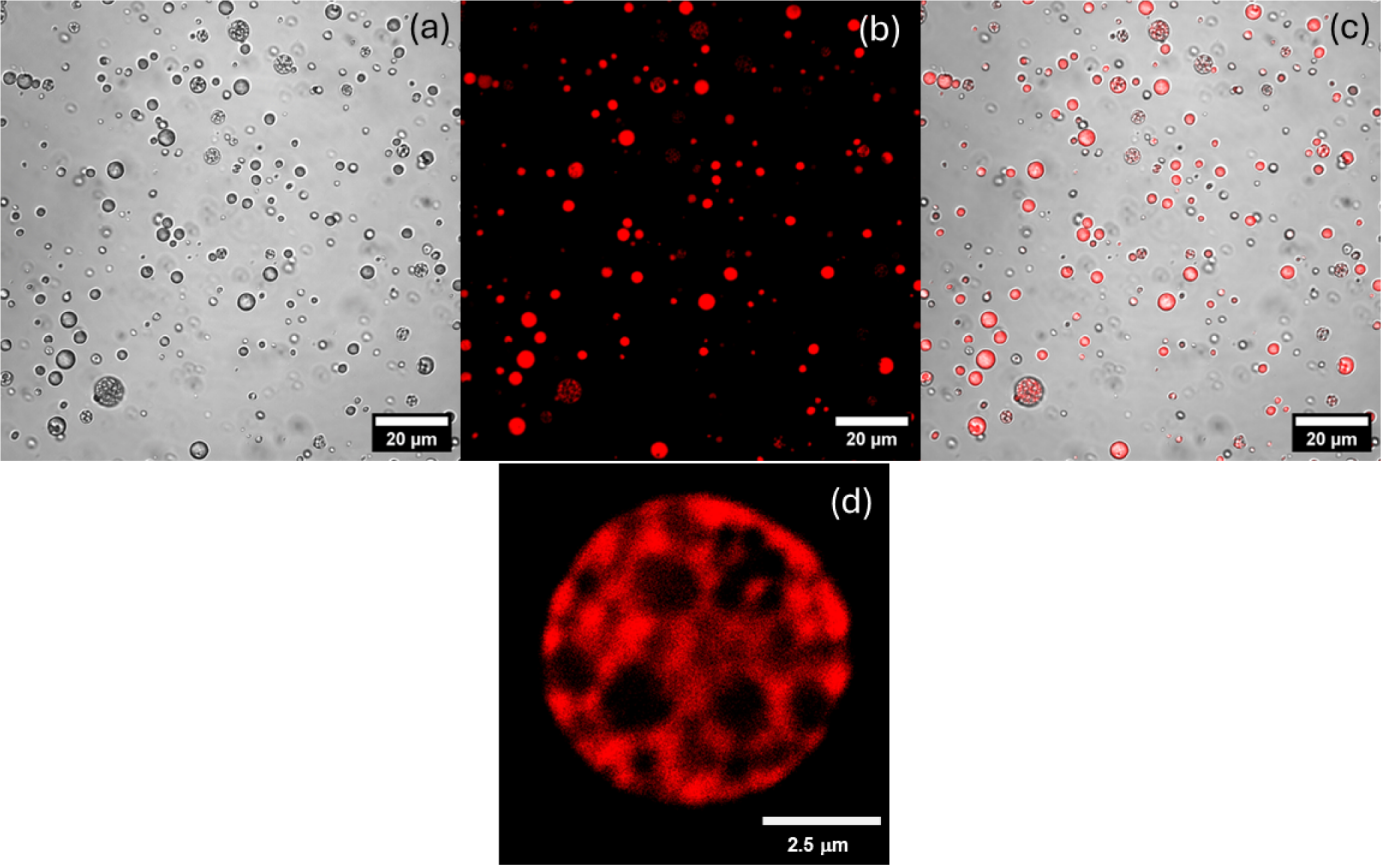
Confocal microscopy images for 0.1 wt % Fmoc-Pam_2_CysOH stained with 3 × 10^–5^ wt % Nile red. (a) Bright-field image, (b) fluorescence image, (c) merged image, and (d) enlarged image of a perforated vesicle.

The CAC was first determined from Nile red fluorescence assays. As for the other conjugates, the CAC can be detected from variations in fluorescence intensity (*I*/*I*_0_) and blue shifts in peak position, as evident from the data in Figure S1F (original spectra in Figure S1E). The CAC was found to be (0.018 ± 0.003) wt %, slightly lower than the values for the other conjugates, as expected due to the presence of the hydrophobic Fmoc group, which facilitates aggregation at a lower concentration. Confocal microscopy was performed on solutions of Fmoc-Pam_2_CysOH rehydrated from films using Nile red as a fluorescent probe. Confocal microscopy images such as those shown in Figure 5 indicate that Fmoc-Pam_2_CysOH self-assembles into vesicle-like structures with some vesicles having an external structure (Figure 5). The size of the vesicles is consistent with the hydrodynamic radius distribution from DLS shown in Figure S3, with *R*_H_ in the range of several hundreds of nanometers for the major population of aggregates. Additional confocal fluorescence microscopy images obtained using Rhodamine B confirmed these findings, and a population of vesicles with perforated/compound structures was also observed (Figure S6). We propose that these may result from an osmotic pressure difference between the vesicle interior and the bulk solution, leading to partial collapse of some (the minority of) vesicles. Alternatively, they may be surface raft-like structures. Dynamic light scattering confirms that the vesicle size distribution is not affected by the addition of Rhodamine B (Figure S7). SAXS data for Fmoc-Pam_2_CysOH indicate a multilamellar structure (Figure S8), in contrast to the unilamellar vesicles formed by Pam_2_CysOH and Pam_3_CysOH. The *d*-spacing is 36.7 Å, which is reasonable considering a bilayer of palmitoyl (C_16_) chains, with the Fmoc group also likely to be within the hydrophobic membrane interior. In addition to the two orders of reflection from the multilamellar structure, there is a broad peak centered at *q* = 0.25 Å^–1^ which is most likely due to a population of unilamellar vesicles, although it could also be a diffuse scattering feature arising from membrane perforations.^39^

The CD spectrum of Fmoc-Pam_2_CysOH shown in SIFigure 9 indicates a lack of defined secondary structure, consistent with vesicle formation. However, there is a notable peak at 262 nm, which is due to the absorption of Fmoc at this wavelength, as shown in the absorption spectrum also plotted, and consistent with prior reports.^40−42^ In fact, Fmoc peptides exhibit fluorescence when excited at a suitable wavelength (here λ = 268 nm), and this can be used to detect aggregation events which lead to a shift in fluorescence intensity and wavelength.^42^Figure S10 shows the measured fluorescence spectra for a concentration series along with a plot of the concentration dependence of the peak intensity, which shows a discontinuity that signals a critical aggregation concentration (CAC) at 0.01 wt % for Fmoc-Pam_2_CysOH, in good agreement with the value from the Nile red fluorescence assays (Figure S1F).

**Figure 6:**
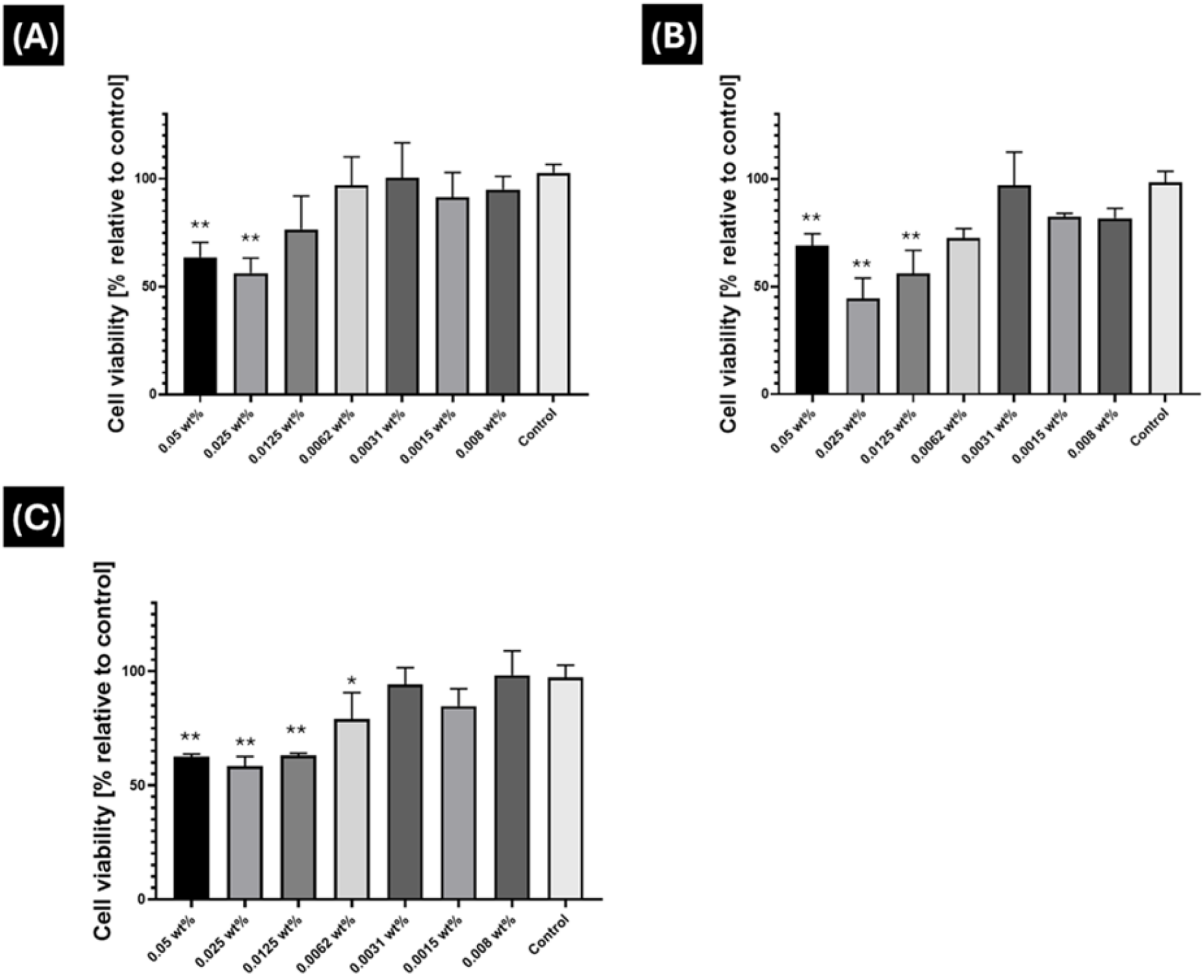
Concentration-dependent cytotoxicity from MTT assays on HEK293T cells (after 72 h) of (A) Pam_2_CysOH, (B) Pam_2_CysSK_4_, and (C) Pam_3_CysSK_4_. The tests were repeated in triplicate (*n* = 3), and the statistical analysis was performed using ANOVA with Bonferroni correction for multiple comparisons. * *p* ≤ 0.05. ** *p* ≤ 0.001.

To be useful in biochemical analyses or potentially as TLR2/6 agonists, the Pam_n_Cys-conjugates should be cytocompatible. Due to very limited solubility in media, Pam_3_CysOH is excluded from the following bioactivity studies. The cytocompatibility of the remaining three conjugates was determined by MTT assays (using HEK 293T cells), a technique used to measure the mitochondrial activity of cells, which serves as an overall cell health indicator.^43^ The data in Figure 6 show that Pam_2_CysOH is cytocompatible at low concentrations, but there is a significant decrease in cell viability when compared to the control at concentrations above 0.025 wt % (Figure 6A), while Pam_2_CysSK_4_ shows cytotoxicity above a slightly lower concentration (0.0125 wt %, Figure 6B). Pam_3_CysSK_4_ was only tolerated below 0.0062 wt % (Figure 6C). Thus, Pam_2_CysOH shows enhanced cytocompatibility compared to its corresponding Pam_n_CysSK_4_ analogues.

Since Pam_2_CysSK_4_ and especially Pam_3_CysSK_4_ are established model TLR agonist lipopeptides,^9,21,26,27^ stimulating TLR2 in particular,^9,21,26^ but also TLR6 for Pam_2_ (diacyl)-based lipopeptides,^9,44−49^ we compared the agonist activity of these two lipopeptides with the conjugate Pam_2_CysOH. We used commercially available HEK-blue TLR2/6 cells which produce secreted alkaline phosphatase (SEAP) in the presence of an agonist of either TLR2 or TLR6. SEAP reacts with the resazurin dye Quanti-Blue in the detection media and can be quantified by spectrophotometry in the wavelength range of 620 ∼ 650 nm.^50^ The SEAP assays extend for comparison up to concentrations of 0.05 wt %, at which a significant reduction in cytocompatibility was observed; however, it should be noted that even under these conditions, typically 50% of cells or more were still viable (Figure 6).

**Figure 7:**
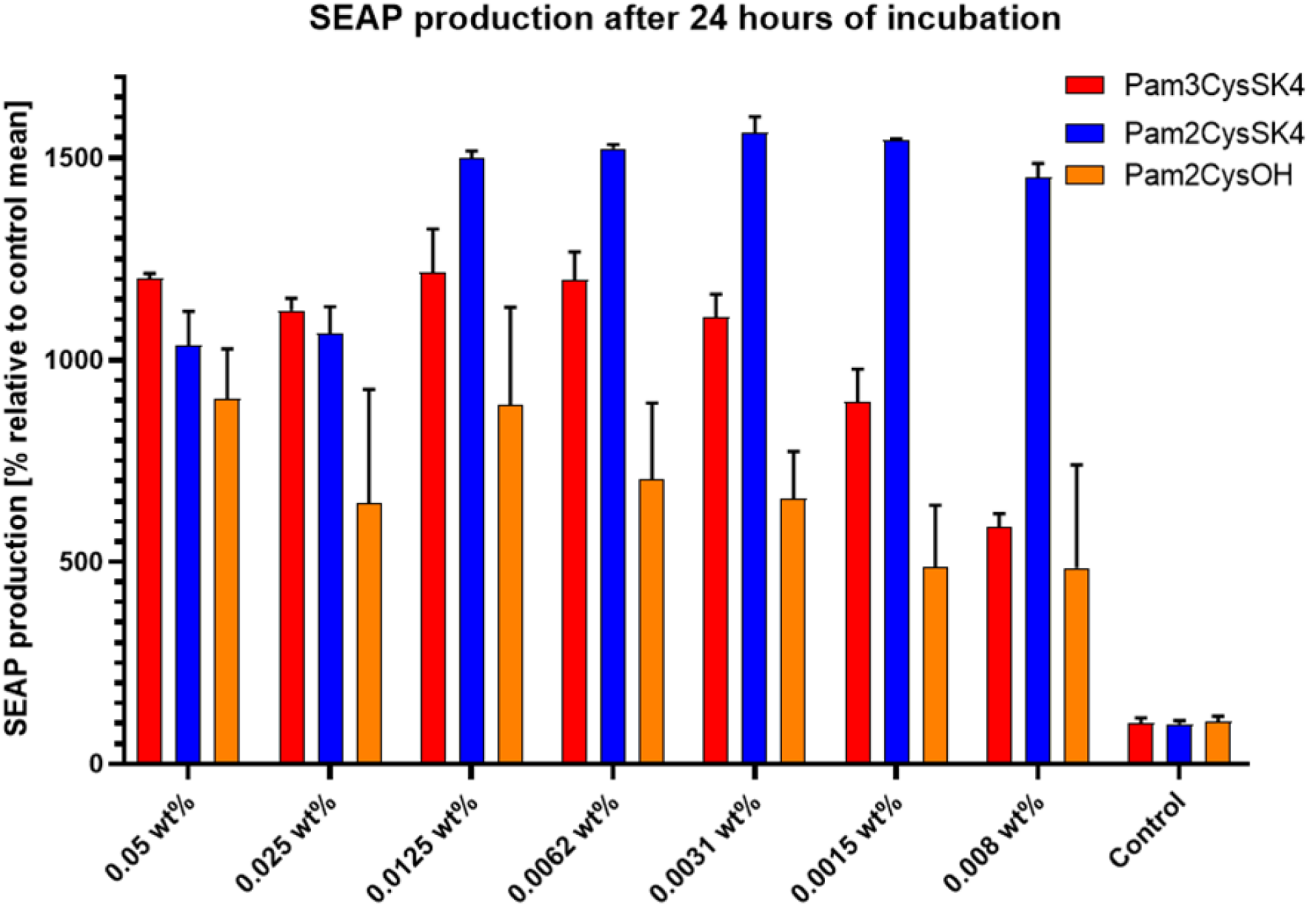
Quantification of the secreted alkaline phosphatase (SEAP) production in HEK293T cells stimulated by different conjugates, as indicated. The control was DMEM only. The data were analyzed by ANOVA with Bonferroni correction for multiple comparisons, *n* = 3. * *p* ≤ 0.05. ** *p* ≤ 0.001.

The data presented in Figure 7 and Table 1 show that both Pam_2_CSK_4_ and Pam_3_CSK_4_ stimulate a 10-fold or greater increase in the expression of SEAP by HEK-Blue cells, even at dilute concentrations in the case of Pam_2_CSK_4_. The new conjugate Pam_2_CysOH also shows some agonist activity, exhibiting an ∼ 8-fold increase at 0.0125 wt %, although it also presented a larger variation in the amount of SEAP induced when compared to the two CysSK_4_ lipopeptides. The data show that the two Pam_2_-based conjugates stimulated greater TLR2/6 activation, as expected, given the strong binding of diacylated lipopeptides (and here, the conjugate Pam_2_CysOH) to TLR2-TLR6 heterodimers that activate nuclear factor κB (NF-κB) and activator protein AP-1, leading to SEAP.

**Table 1:** Mean and Standard Deviation of the SEAP Production for Conjugates at Different Concentrations

Concentration	Pam_2_CysOH	Pam_2_CysSK_4_	Pam_3_CysOH	Pam_3_CysSK_4_
0.05 wt %	901 ± 89%	1034 ± 61%	134 ± 17%	1189 ± 9%
0.025 wt %	644 ± 202%	1063 ± 49%	105 ± 13%	1098 ± 22%
0.0125 wt %	888 ± 173%	1499 ± 13%	121 ± 8%	1118 ± 76%
0.0062 wt %	702 ± 136%	1523 ± 7%	127 ± 2%	1128 ± 49%
0.0031 wt %	656 ± 84%	1561 ± 29%	110 ± 5%	1049 ± 40%
0.0015 wt %	485 ± 110%	1544 ± 2%	104 ± 3%	811 ± 58%
0.008 wt %	486 ± 182%	1452 ± 24%	101 ± 2%	554 ± 24%
Control	104 ± 9%	98 ± 6%	97 ± 2%	99 ± 9%

## Conclusions

We examined the self-assembly of Pam_2_Cys and Pam_3_Cys-based compounds, comparing the parent Pam_2_CysOH and Pam_3_CysOH with the bioactive TLR-agonist lipopeptides Pam_2_CSK_4_ and Pam3CSK_4_. All examined conjugates exhibit a critical aggregation concentration, detected through Nile red fluorescence assays. SAXS, cryo-TEM, and fluorescence microscopy imaging indicate that Pam_2_CysOH and Pam_3_CysOH both form unilamellar vesicles, although the vesicles are smaller for the former, probably due to increased membrane fluidity. This may result from the lower number of lipid chains for Pam_2_CysOH (reduced steric constraints on lipid chain packing) and/or changes in the charge at the vesicle surface which will contain amino and carboxyl functions for Pam_2_CysOH (zwitterionic) but only carboxyl groups for Pam_3_CysOH or Fmoc-Pam_2_CysOH, leading to a negative charge in the latter two cases. These nanostructures are very different from those of Pam_2_CSK_4_ which forms spherical micelles and Pam_3_CSK_4_ which forms mainly wormlike micelles (with a population of globules)

Comparing the self-assemblies of Pam_2_CysOH and Fmoc-Pam_2_CysOH, we find that the former forms an extensive population of small vesicles, whereas the latter forms isolated large vesicles, some of which show a perforated/compound structure. It is clear that the addition of the Fmoc influences self-assembly, probably due to its influence on membrane fluidity, which may be reduced compared to that of Pam_2_CysOH and Pam_3_CysOH. The vesicles of Fmoc-Pam_2_CysOH predominantly exhibit a multilamellar structure rather than the unilamellar vesicles of Pam_2_CysOH and Pam_3_CysOH.

The vesicles formed by Pam_2_CysOH, Pam_3_CysOH, and Fmoc-Pam_2_Cys will comprise a bilayer membrane with the hydrophobic lipid (or mixed Fmoc/lipid) interior and a carboxyl (plus amino for Pam_2_CysOH) coating on the inner and outer vesicle surfaces. The carboxyl groups could serve as tags for further functionalization of the vesicles for future applications, such as biocatalysis or encapsulation/delivery systems.

The nano/micro-sized vesicles show a population of some virus-sized particles (and in the case of Fmoc-Pam_2_CysOH particles with an external structure resembling perforated or compound vesicles). Conjugates Pam_2_CysOH, Pam_2_CysSK_4_, and Pam_3_CysSK_4_ show good cytocompatibility at sufficiently low concentrations, with slightly better cell compatibility at high concentrations for Pam_2_CysOH conjugates compared to the Pam_n_CSK_4_ analogues. This may be due to the expected cytotoxicity of cationic tetralysine sequences in the Pam_n_CSK_4_ lipopeptides. Pam_2_CysOH shows good TLR2/6 activation through a HEK-Blue secreted alkaline phosphatase dye assay, with up to an 8-fold increase in SEAP production. Our results suggest that due to its vesicular structure (and population of virus-sized structures), Pam_2_CysOH is of great interest for further studies on immune system stimulation, both *in vitro* and *in vivo*.

## Supporting Information Available

Nile red fluorescence spectra, confocal microscopy images, dynamic light scattering data, CD spectra, and cryo-TEM images for Pam_2_CysSK_4_ and Pam_3_CysSK_4_; confocal microscopy, DLS and SAXS data, CD and absorbance spectra, and CAC assay for Fmoc-Pam_2_CysOH solutions (PDF)

## References

[ref1] Cavalli S.; Albericio F.; Kros A. Amphiphilic peptides and their cross-disciplinary role as building blocks for nanoscience. Chem. Soc. Rev. 2010, 39 (1), 241–263. 10.1039/B906701A. 20023851

[ref2] Cui H. G.; Webber M. J.; Stupp S. I. Self-Assembly of Peptide Amphiphiles: From Molecules to Nanostructures to Biomaterials. Biopolymers 2010, 94 (1), 1–18. 10.1002/bip.21328. 20091874 PMC2921868

[ref3] Matson J. B.; Zha R. H.; Stupp S. I. Peptide self-assembly for crafting functional biological materials. Curr. Opin. Solid State Mater. Sci. 2011, 15, 225–235. 10.1016/j.cossms.2011.08.001. 22125413 PMC3224089

[ref4] Matson J. B.; Stupp S. I. Self-assembling peptide scaffolds for regenerative medicine. Chem. Commun. 2012, 48 (1), 26–33. 10.1039/C1CC15551B. PMC3355058 22080255

[ref5] Webber M. J.; Berns E. J.; Stupp S. I. Supramolecular Nanofibers of Peptide Amphiphiles for Medicine. Isr. J. Chem. 2013, 53 (8), 530–554. 10.1002/ijch.201300046. 24532851 PMC3922220

[ref6] Arslan E.; Garip I. C.; Gulseren G.; Tekinay A. B.; Guler M. O. Bioactive Supramolecular Peptide Nanofibers for Regenerative Medicine. Adv. Healthcare Mater. 2014, 3 (9), 1357–1376. 10.1002/adhm.201300491. 24574311

[ref7] Hamley I. W. Lipopeptides: From self-assembly to bioactivity. Chem. Commun. 2015, 51, 8574–8583. 10.1039/C5CC01535A. 25797909

[ref8] Hutchinson J. A.; Burholt S.; Hamley I. W. Peptide hormones and lipopeptides: from self-assembly to therapeutic applications. J. Pept. Sci. 2017, 23, 82–94. 10.1002/psc.2954. 28127868 PMC5324658

[ref9] Hamley I. W. Lipopeptides for Vaccine Development. Bioconjugate Chem. 2021, 32, 1472–1490. 10.1021/acs.bioconjchem.1c00258. PMC8382226 34228433

[ref10] Vicente-Garcia C.; Colomer I. Lipopeptides as tools in catalysis, supramolecular, materials and medicinal chemistry. Nature Rev. Chem. 2023, 7 (10), 710–731. 10.1038/s41570-023-00532-8. 37726383

[ref11] Löwik D. W. P. M.; van Hest J. C. M. Peptide based amphiphiles. Chem. Soc. Rev. 2004, 33, 234–245. 10.1039/B212638A. 15103405

[ref12] Versluis F.; Marsden H. R.; Kros A. Power struggles in peptide-amphiphile nanostructures. Chem. Soc. Rev. 2010, 39 (9), 3434–3444. 10.1039/b919446k. 20644886

[ref13] Hamley I. W. Self-Assembly of Amphiphilic Peptides. Soft Matter 2011, 7, 4122–4138. 10.1039/c0sm01218a.

[ref14] Trent A.; Marullo R.; Lin B.; Black M.; Tirrell M. Structural properties of soluble peptide amphiphile micelles. Soft Matter 2011, 7 (20), 9572–9582. 10.1039/c1sm05862b.

[ref15] Hendricks M. P.; Sato K.; Palmer L. C.; Stupp S. I. Supramolecular Assembly of Peptide Amphiphiles. Acc. Chem. Res. 2017, 50 (10), 2440–2448. 10.1021/acs.accounts.7b00297. 28876055 PMC5647873

[ref16] Santoso S.; Hwang W.; Hartman H.; Zhang S. Self-assembly of surfactant-like peptides with variable glycine tails to form nanotubes and nanovesicles. Nano Lett. 2002, 2 (7), 687–691. 10.1021/nl025563i.

[ref17] van Hell A. J.; Costa C. I. C. A.; Flesch F. M.; Sutter M.; Jiskoot W.; Crommelin D. J.; Hennink W. E.; Mastrobattista E. Self-assembly of recombinant amphiphilic oligopeptides into vesicles. Biomacromolecules 2007, 8, 2753–2761. 10.1021/bm0704267. 17696394

[ref18] Adler-Abramovich L.; Kol N.; Yanai I.; Barlam D.; Shneck R. Z.; Gazit E.; Rousso I. Self-Assembled Organic Nanostructures with Metallic-Like Stiffness. Angew. Chem., Int. Ed. 2010, 49 (51), 9939–9942. 10.1002/anie.201002037. 20878815

[ref19] Felip-Leon C.; Galindo F.; Miravet J. F.; Castelletto V.; Hamley I. W. Thermally Regulated Reversible Formation of Vesicle-Like Assemblies by Hexaproline Amphiphiles. J. Phys. Chem. B 2017, 121 (31), 7443–7446. 10.1021/acs.jpcb.7b06167. 28719210

[ref20] Bernal-Martínez A. M.; Bedrina B.; Angulo-Pachón C. A.; Galindo F.; Miravet J. F.; Castelletto V.; Hamley I. W. pH-Induced conversion of bolaamphiphilic vesicles to reduction-responsive nanogels for enhanced Nile Red and Rose Bengal delivery. Colloid Surf. B 2024, 242, 114072 10.1016/j.colsurfb.2024.114072. 39024718

[ref21] Lu B. L.; Williams G. M.; Brimble M. A. TLR2 agonists and their structure–activity relationships. Org. Biomol. Chem. 2020, 18, 5073–5094. 10.1039/D0OB00942C. 32582902

[ref22] Braun V. Covalent Lipoprotein from Outer Membrane of *Escherichia coli*. Biochim. Biophys. Acta (BBA) 1975, 415 (3), 335–377. 10.1016/0304-4157(75)90013-1. 52377

[ref23] Reitermann A.; Metzger J.; Wiesmuller K. H.; Jung G.; Bessler W. G. Lipopeptide Derivatives of Bacterial Lipoprotein Constitute Potent Immune Adjuvants Combined with or Covalently Coupled to Antigen or Hapten. Biol. Chem. 1989, 370 (4), 343–352. 10.1515/bchm3.1989.370.1.343. 2757794

[ref24] Hantke K.; Braun V. Covalent Binding of Lipid to Protein - Diglyceride and Amide-Linked Fatty-Acid at N-Terminal end of Murein-Lipoprotein of *Escherichia coli* Outer Membrane. Eur. J. Biochem. 1973, 34 (2), 284–296. 10.1111/j.1432-1033.1973.tb02757.x. 4575979

[ref25] Bessler W.; Resch K.; Hancock E.; Hantke K. Induction of Lymphocyte Proliferation and Membrane Changes by Lipopeptide Derivatives of Lipoprotein from Outer Membrane of Escherichia Coli. Z. Immunitatsforschung-Immunobiol. 1977, 153 (1), 11–22. 10.1016/S0340-904X(77)80023-7. 325934

[ref26] Moyle P. M.; Toth I. Self-adjuvanting lipopeptide vaccines. Curr. Med. Chem. 2008, 15 (5), 506–516. 10.2174/092986708783503249. 18289006

[ref27] Zom G. G. P.; Khan S.; Filippov D. V.; Ossendorp F. TLR Ligand-Peptide Conjugate Vaccines: Toward Clinical Application. In Advances in Immunology, Melief C. J. M., Ed.; Elsevier Academic Press Inc.: San Diego, 2012; Vol. 114, pp. 177–201. 10.1016/B978-0-12-396548-6.00007-X 22449782

[ref28] Hamley I. W.; Kirkham S.; Dehsorkhi A.; Castelletto V.; Reza M.; Ruokolainen J. Toll-like Receptor Agonist Lipopeptides Self-Assemble into Distinct Nanostructures. Chem. Commun. 2014, 50, 15948–15951. 10.1039/C4CC07511K. 25382300

[ref29] Zhao L.; Tu Y. S.; Fang H. P.; Hamley I. W.; Wang Z. W. Self-Assembled Micellar Structures of Lipopeptides with Variable Number of Attached Lipid Chains Revealed by Atomistic Molecular Dynamics Simulations. J. Phys. Chem. B 2018, 122 (41), 9605–9615. 10.1021/acs.jpcb.8b07877. 30253107

[ref30] Castelletto V.; Seitsonen J.; de Mello L.; Hamley I. W. Interaction of Arginine-Rich Surfactant-Like Peptide Nanotubes with Liposomes. Biomacromolecules 2024, 25, 7410–7420. 10.1021/acs.biomac.4c01072. 39469728 PMC11558666

[ref31] Provencher S. W. CONTIN: A general purpose constrained regularization program for inverting noisy linear algebraic and integral equations. Comput. Phys. Commun. 1982, 27, 229 10.1016/0010-4655(82)90174-6.

[ref32] Cowieson N. P.; Edwards-Gayle C. J. C.; Inoue K.; Khunti N. S.; Doutch J.; Williams E.; Daniels S.; Preece G.; Krumpa N. A.; Sutter J. P.; et al. Beamline B21: high-throughput small-angle X-ray scattering at Diamond Light Source. J. Synchrotron Radiat. 2020, 27, 1438–1446. 10.1107/S1600577520009960. 32876621 PMC7467336

[ref33] Greenspan P.; Mayer E. P.; Fowler S. D. Nile red - A Selective Fluorescent Stain for Intracellular Lipid Droplets. J. Cell Biol. 1985, 100 (3), 965–973. 10.1083/jcb.100.3.965. 3972906 PMC2113505

[ref34] Sackett D. L.; Wolff J. Nile red as a Polarity-Sensitive Fluorescent Probe of Hydrophobic Protein Surfaces. Anal. Biochem. 1987, 167 (2), 228–234. 10.1016/0003-2697(87)90157-6. 3442318

[ref35] Hawe A.; Sutter M.; Jiskoot W. Extrinsic fluorescent dyes as tools for protein characterization. Pharm. Res. 2008, 25 (7), 1487–1499. 10.1007/s11095-007-9516-9. 18172579 PMC2440933

[ref36] Greenspan P.; Fowler S. D. Spectrofluorometric Studies of the Lipid Probe, Nile Red. J. Lipid Res. 1985, 26 (7), 781–789. 10.1016/S0022-2275(20)34307-8. 4031658

[ref37] Edwards-Gayle C. J. C.; Castelletto V.; Hamley I. W.; Barrett G.; Greco F.; Hermida-Merino D.; Rambo R.; Seitsonen J.; Ruokolainen J. Self-assembly, Antimicrobial Activity and Membrane Interactions of Arginine-capped Peptide Bola-amphiphiles ACS Appl. Bio. Mater. 2019, 2, 2208–2218. 10.1021/acsabm.9b00172. PMC6537463 31157325

[ref38] Hamley I. W. Self-Assembly, Bioactivity and Nanomaterials Applications of Peptide Conjugates with Bulky Aromatic Terminal Groups. ACS Appl. Bio Mater. 2023, 6, 384–409. 10.1021/acsabm.2c01041. PMC9945136 36735801

[ref39] Hamley I. W. Diffuse scattering from lamellar structures. Soft Matter 2022, 18 (4), 711–721. 10.1039/D1SM01758F. 35014650

[ref40] Ryan D. M.; Doran T. M.; Nilsson B. L. Stabilizing self-assembled Fmoc-F-5-Phe hydrogels by co-assembly with PEG-functionalized monomers. Chem. Commun. 2011, 47 (1), 475–477. 10.1039/C0CC02217A. 20936201

[ref41] Zou Y.; Razmkhah K.; Chmel N. P.; Hamley I. W.; Rodger A. Spectroscopic signatures of an Fmoc-tetrapeptide, Fmoc and fluorene. RSC Adv. 2013, 3 (27), 10854–10858. 10.1039/c3ra41979g.

[ref42] Castelletto V.; de Mello L.; da Silva E. R.; Seitsonen J.; Hamley I. W. Comparison of the self-assembly and cytocompatibility of conjugates of Fmoc (9-fluorenylmethoxycarbonyl) with hydrophobic, aromatic, or charged amino acids. J. Pept. Sci. 2024, 30, e3571 10.1002/psc.3571. 38374800

[ref43] van Meerloo J.; Kaspers G. J. L.; Cloos J. Cell Sensitivity Assays: The MTT Assay In Cancer Cell Culture: Methods Protocols; Humana Press, 2011, 731, PP. 237–245. DOI: 10.1007/978-1-61779-080-5_20. 21516412

[ref44] Akira S.; Takeda K. Toll-like receptor signalling. Nat. Rev. Immunol. 2004, 4 (7), 499–511. 10.1038/nri1391. 15229469

[ref45] Akira S.; Uematsu S.; Takeuchi O. Pathogen recognition and innate immunity. Cell 2006, 124, 783–801. 10.1016/j.cell.2006.02.015. 16497588

[ref46] Oliveira-Nascimento L.; Massari P.; Wetzler L. M. The role of TLR2 in infect on and immunity. Front. Immunol. 2012, 3, 79 10.3389/fimmu.2012.00079. 22566960 PMC3342043

[ref47] Irvine K. L.; Hopkins L. J.; Gangloff M.; Bryant C. E. The molecular basis for recognition of bacterial ligands at equine TLR2, TLR1 and TLR6. Vet. Res. 2013, 44, 50 10.1186/1297-9716-44-50. 23826682 PMC3716717

[ref48] Castelletto V.; Kirkham S.; Hamley I. W.; Kowalczyk R.; Rabe M.; Reza M.; Ruokolainen J. Self-Assembly of the Toll-Like Receptor Agonist Macrophage-Activating Lipopeptide MALP-2 and of Its Constituent Peptide. Biomacromolecules 2016, 17 (2), 631–640. 10.1021/acs.biomac.5b01573. 26752598

[ref49] Parra-Izquierdo I.; Lakshmanan H. H. S.; Melrose A. R.; Pang J. Q.; Zheng T. J.; Jordan K. R.; Reitsma S. E.; McCarty O. J. T.; Aslan J. E. The Toll-Like Receptor 2 Ligand Pam2CSK4 Activates Platelet Nuclear Factor-κB and Bruton’s Tyrosine Kinase Signaling to Promote Platelet-Endothelial Cell Interactions. Front. Immunol. 2021, 12, 729951 10.3389/fimmu.2021.729951. 34527000 PMC8435771

[ref50] Hu Z. Y.; Zhang T.; Jiang S. S.; Yin H. Protocol for evaluation and validation of TLR8 antagonists in HEK-Blue cells via secreted embryonic alkaline phosphatase assay. STAR Protoc. 2022, 3 (1), 101061 10.1016/j.xpro.2021.101061. 35005643 PMC8715332

