# Supplementary material for: Self-Assembly of Toll-Like Receptor (TLR2/6) Agonist Lipidated Amino Acid or Peptide Conjugates: Distinct Morphologies and Bioactivities
Source: Bioconjug Chem. 2025 Apr 2;36(4):792–802. doi: 10.1021/acs.bioconjchem.5c00051 (PMC12006960; doi:10.1021/acs.bioconjchem.5c00051)
Supplement: Supplementary file 1 — bc5c00051_si_001.pdf [file bc5c00051_si_001.pdf]

Supporting Information

Self-Assembly of Toll-Like Receptor (TLR2/6) Agonist Lipidated Amino Acid or Peptide Conjugates: Distinct Morphologies and Bioactivities

Valeria Castelletto,[1] Lucas R. de Mello,[1] Juliane Pelin,[1,2] Ian W Hamley[1,*]

[1] *School of Chemistry, Food Biosciences and Pharmacy, University of Reading, Whiteknights, Reading RG6 6AD, U.K.*

[2] *Currently at Departamento de Ciências Farmacêuticas, Universidade Federal de São Paulo, 09913-030, Diadema, São Paulo, Brazil.*

* Author for correspondence: I.W.Hamley@reading.ac.uk

Contents

S-3… **Fig.S1**. Nile red fluorescence spectra and CAC determination
S-4… **Fig.S2.** Confocal microscopy image showing fibrils and a vesicle for Pam_3CysOH.
S-5… **Fig.S3.** Intensity-average distribution of hydrodynamic radius from dynamic light scattering.
S-6… **Fig.S4**. CD spectra for Pam_2CysSK_4 and Pam_3CysSK_4 (conditions indicated).
S-7… **Fig.S5.** Cryo-TEM images obtained of Pam_2CysSK_4 Pam_3CysSK_4.
S-8… **Fig.S6.** Confocal microscopy images from a solution of 0.01 wt% Fmoc-Pam_2CysOH + 0.0003 wt% Rhodamine B.
S-9… **Fig.S7.** Intensity-averaged distribution of hydrodynamic radius of Fmoc-Pam_2CysOH solutions with and without Rhodamine B, from dynamic light scattering.
S-10…**Fig.S8**. SAXS data from a 2 wt% solution of Fmoc-Pam_2CysOH.
S-11…**Fig.S9**. CD and absorbance spectra from 0.1 wt% solution of Fmoc-Pam_2CysOH.
S-12…**Fig.S10.** Fmoc Fluorescence emission spectra for Fmoc-Pam_2CysOH and concentration dependence of fluorescence intensity showing CAC at the value indicated
S-13…**Table S1**. SAXS data fitted parameters from fits shown in Fig.1.

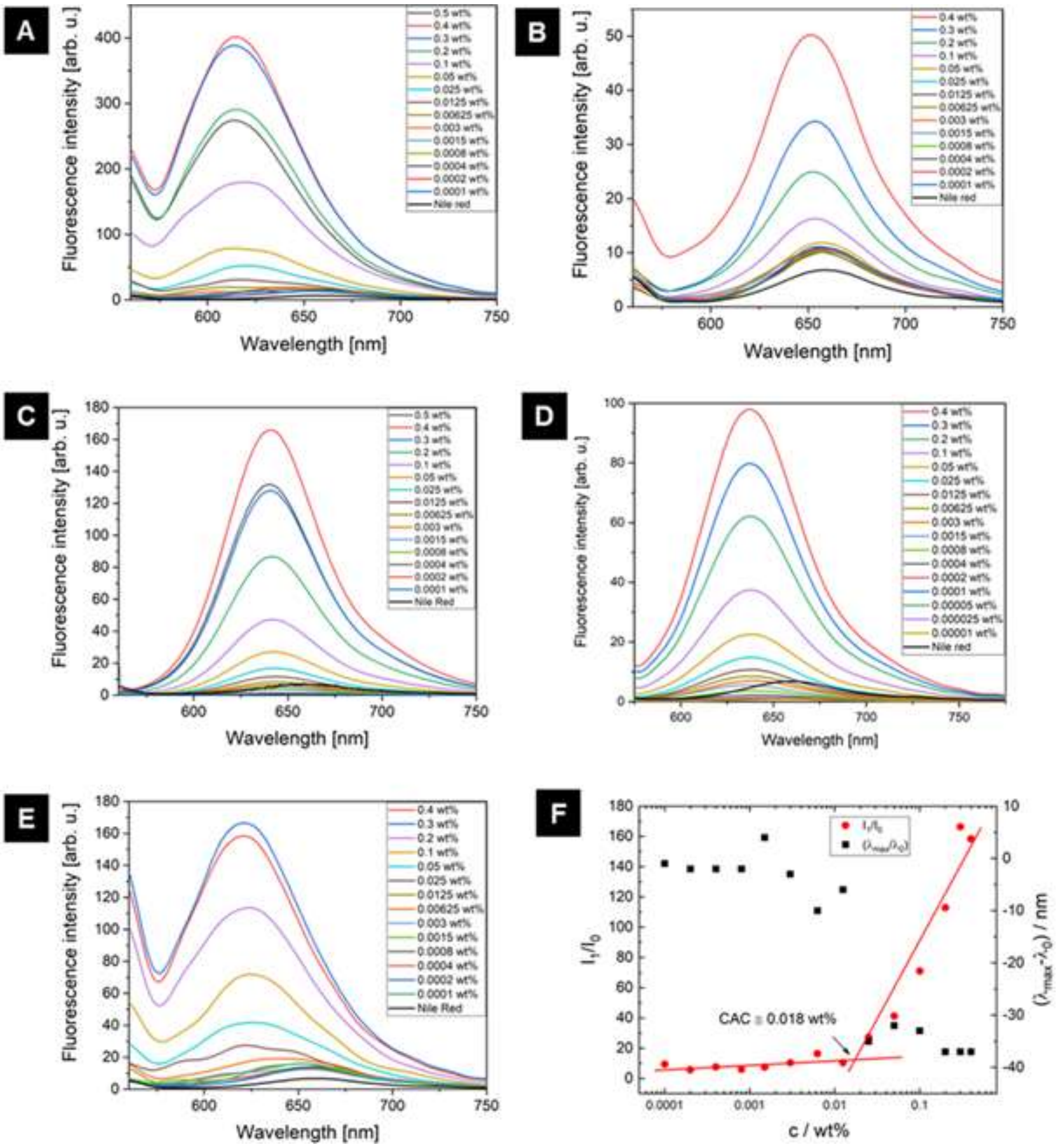

Fig.S1. Nile red fluorescence spectra for (A) Pam_2CysOH, (B) Pam_3CysOH, (C) Pam_2CysSK_4, (D) Pam_3CysSK_4, (E) Fmoc-Pam_2CysOH, (F) CAC determination for Fmoc-Pam_2CysOH. Left axis: I_1/I_0 (red dots) with the CAC; Right axis: blue shift represented by the λ_{max}-λ_0 (black dots).

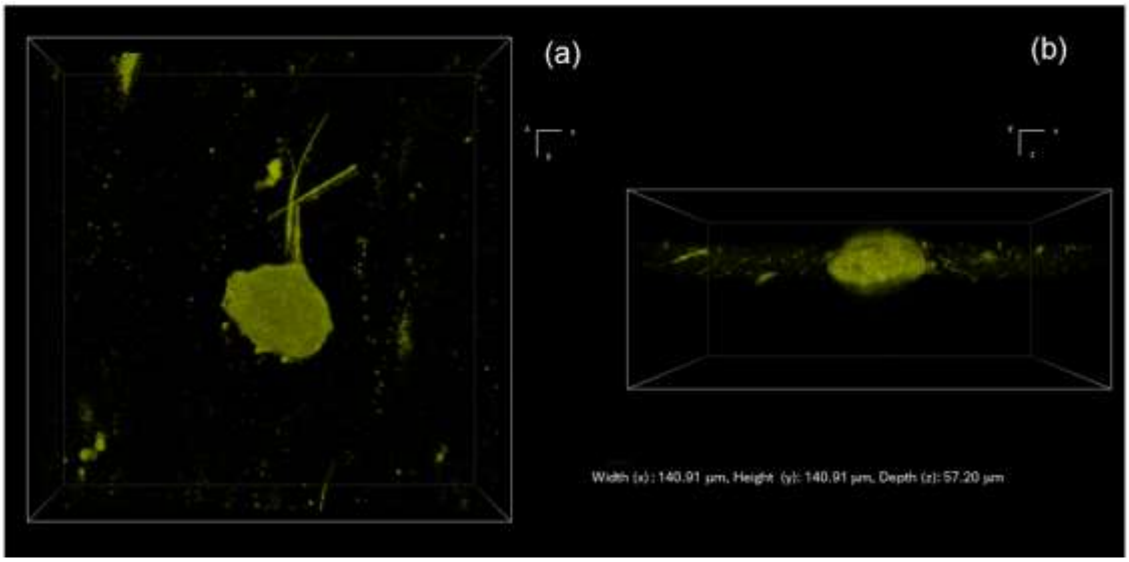

Fig.S2. Confocal microscopy image showing fibrils and a vesicle for Pam_3CysOH: view along (a) the Z- axis or (b) the Y-axis.

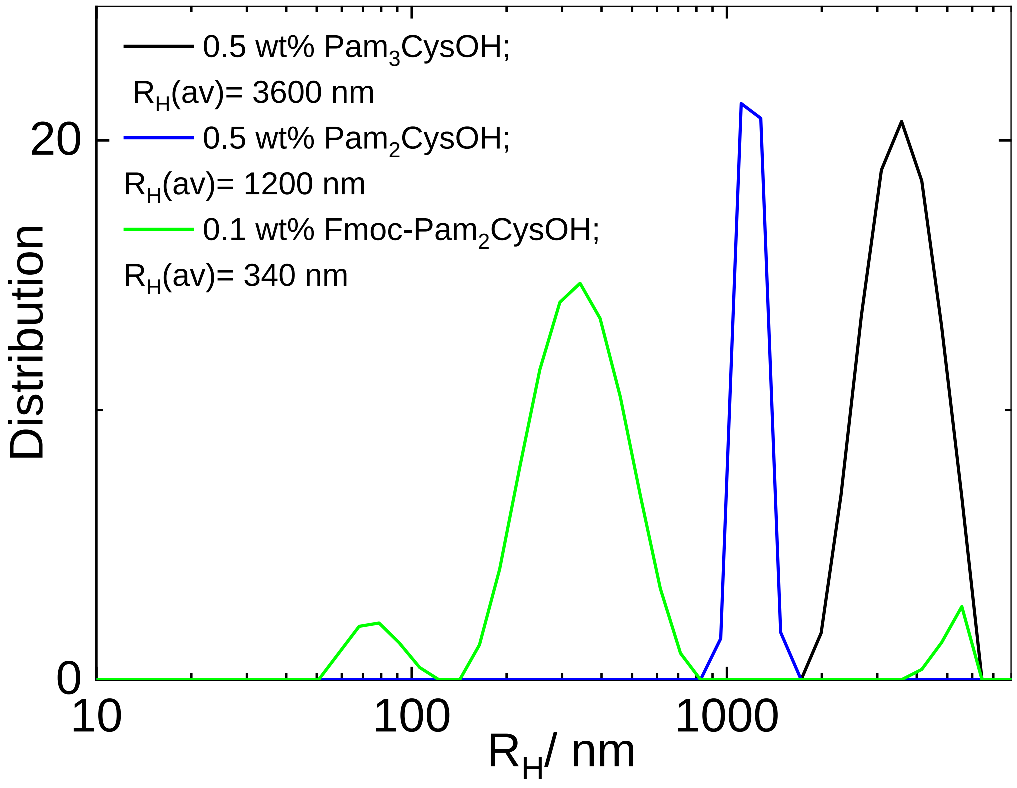

Fig.S3. Intensity-average distribution of hydrodynamic radius for 0.1 wt% Fmoc-Pam_2Cys, 0.5 wt% Pam_2CysOH and 0.5 wt% Pam_3CysOH from dynamic light scattering. Peak average R_H values are indicated.

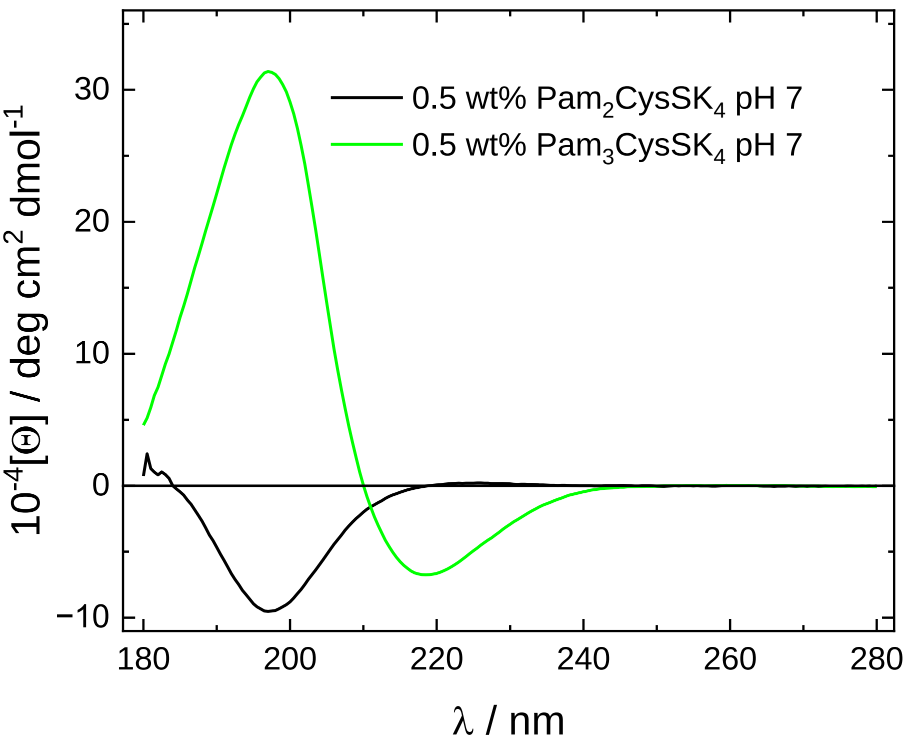

Fig.S4. CD spectra for Pam_2CysSK_4 and Pam_3CysSK_4 (conditions indicated).

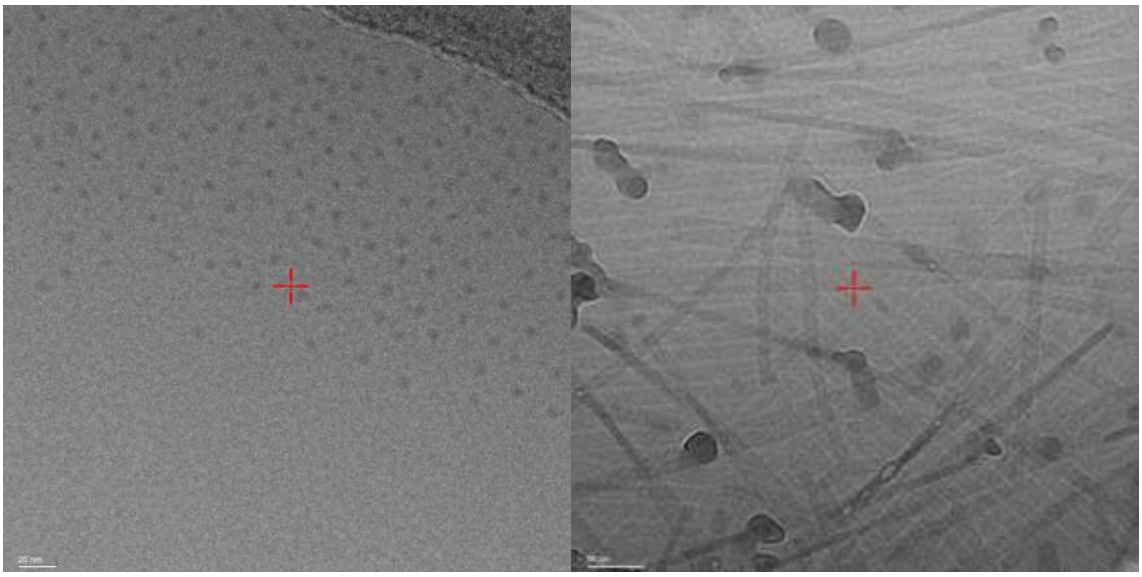

Fig.S5. Cryo-TEM images obtained from 0.5 wt% (pH 7) solutions of (a) Pam_2CysSK_4 and (b) Pam_3CysSK_4.

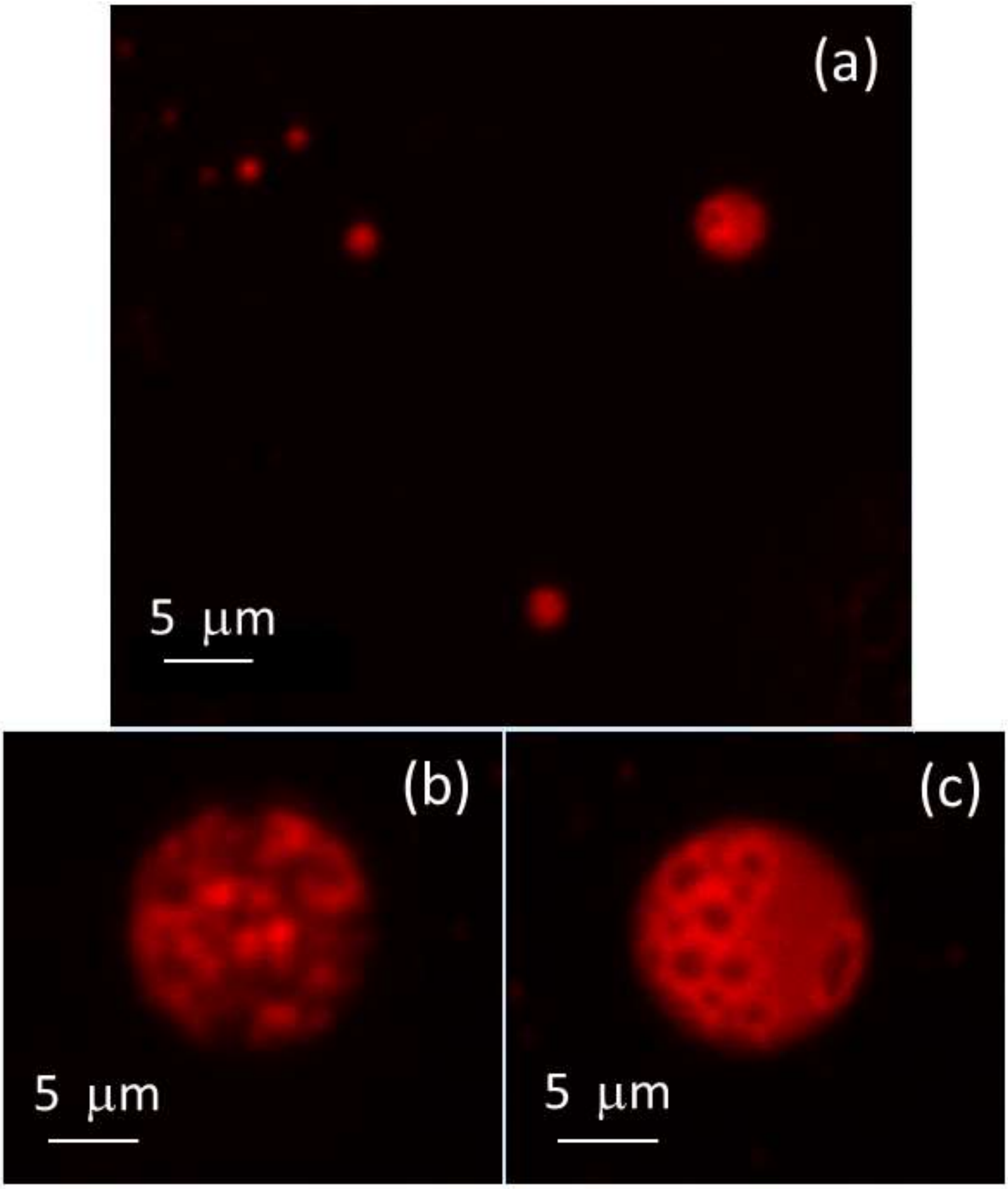

Fig.S6. Confocal microscopy images from a solution of 0.01 wt% Fmoc-Pam_2CysOH + 0.0003 wt% Rhodamine B. The lower image is an enlargement of a vesicle with apparent perforated/compound structure.

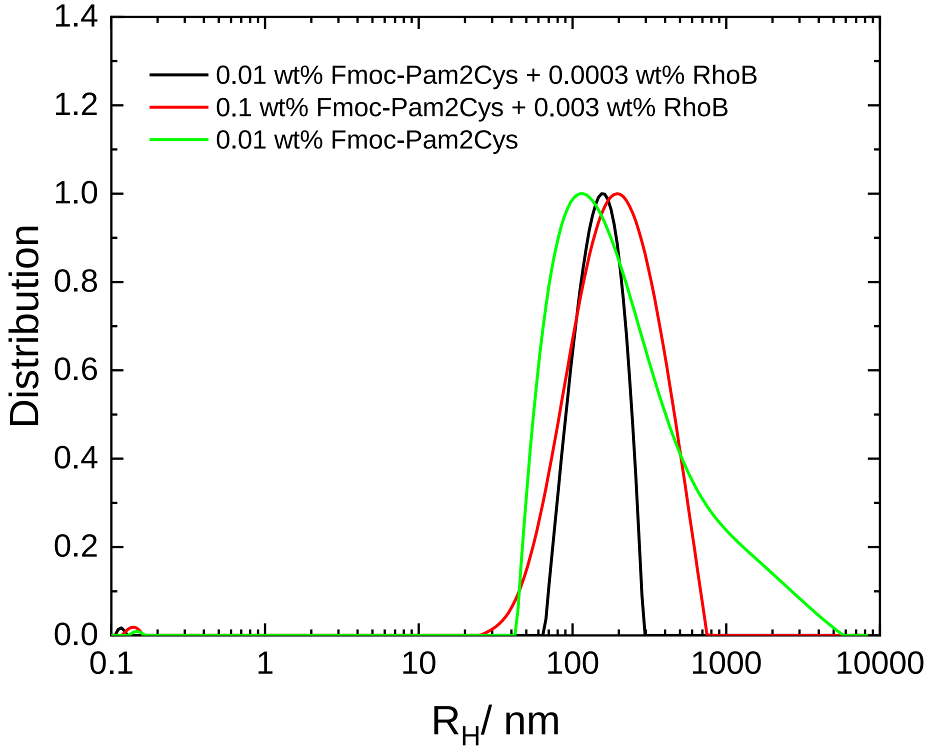

Fig.S7. Intensity-averaged distribution of hydrodynamic radius of Fmoc-Pam$_2$CysOH solutions (conditions shown) with and without Rhodamine B, from dynamic light scattering (data normalized to peak intensity distribution = 1).

.

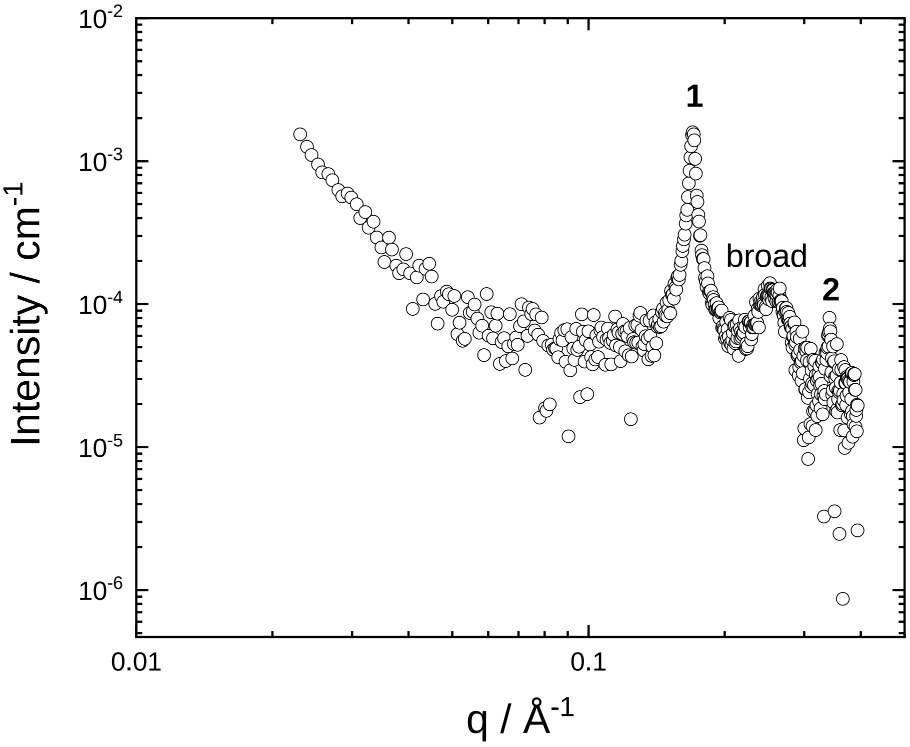

Fig.S8. SAXS data from a 2 wt% solution of Fmoc-Pam$_2$CysOH. First and second order reflections from a multilamellar structure are indicated, along with an additional broad peak discussed in the text.

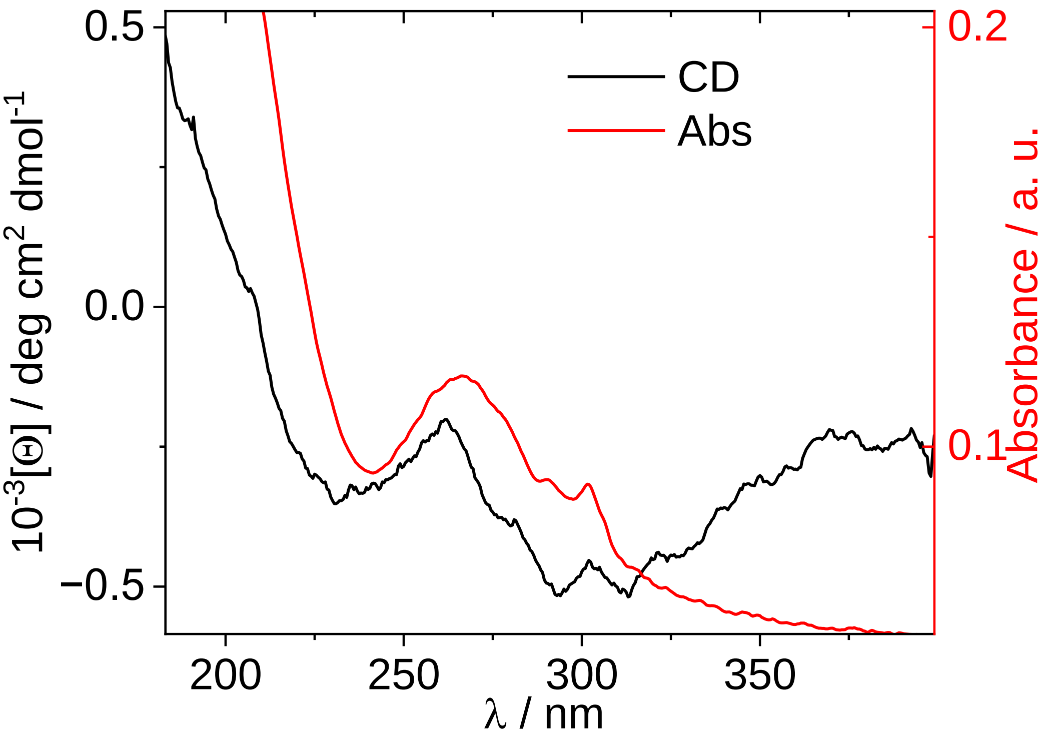

Fig.S9. CD and absorbance spectra from 0.1 wt% solution of Fmoc-Pam_2CysOH.

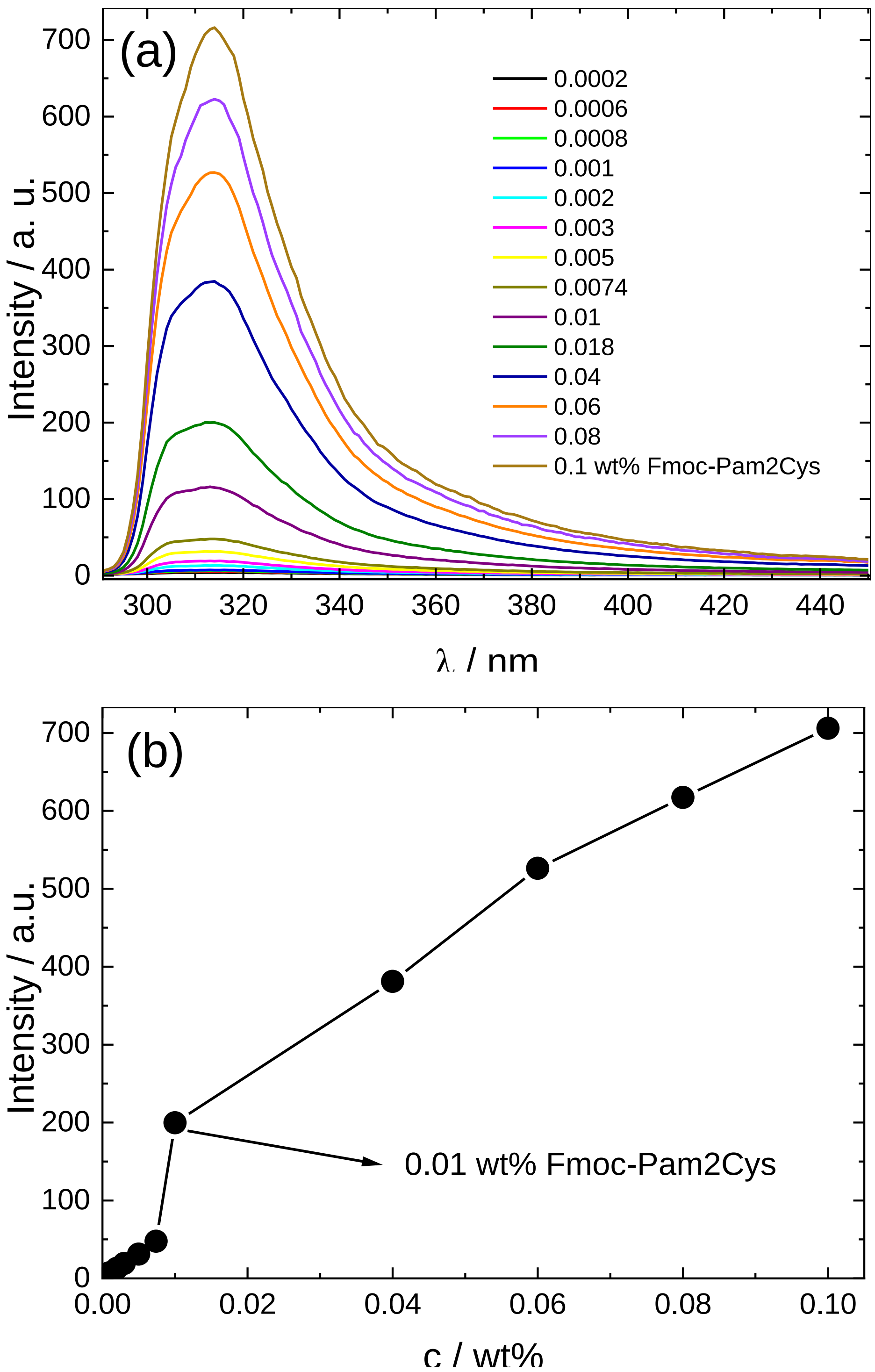

Fig.S10. (a) Fmoc Fluorescence emission spectra for Fmoc-Pam$_2$CysOH at the concentrations shown, (b) Concentration dependence of fluorescence intensity showing CAC at the value indicated.

Table S1. SAXS data fitted parameters from fits shown in Fig.1 obtained using SASfit,[4, 5] and the models detailed. All data for 0.5 wt% samples at the pH values indicated.

**Parameter**	**Pam2CysSK4 pH 7**	**Pam2CysOH pH 7**	**Pam3CysSK4 pH 7**	**Pam3CysOH pH 7**
R_o / Å	44.5			
ΔR / Å	4.8			
R_i / Å	20.9			
μ	-2			
η / cm^{-1}	6.59×10^{-7}			
BG / cm^{-1}	8.4×10^{-4}			
t / Å		44.0	54.0	46.0
Δt / Å		4.5	6.0	4.
η_{out} / cm^{-1}		1.96×10^{-7}	3.46×10^{-7}	2.20×10^{-7}
η_{in} / cm^{-1}		-1.00×10^{-7}	-8.26×10^{-8}	-1.10×10^{-7}
σ_{out} / Å		4.5	5.0	4.1
σ_{in} / Å		6.2	5.0	10.0
D / Å		776.2	831.8	751.3
BG / cm^{-1}		5.0×10^{-4}	5.0×10^{-4}	3.4×10^{-4}

Key:

Core-shell sphere form factor: R_o, outer radius (Gaussian polydispersity ΔR); R_i, inner core radius; μ, scattering contrast of inner core (relative to shell); η, scattering contrast of shell; BG, background.

Gaussian bilayer: layer thickness, t (Gaussian polydispersity Δt); scattering contrast of outer layers η_{out}, and inner layer η_{in}; Gaussian widths σ_{out} and σ_{in} of outer and inner layers respectively; D diameter (width) of layer system (when $D >> t$ as here, it acts as a scaling parameter for the form factor); BG background (fixed).

References

1. V. Castelletto, J. Seitsonen, L. de Mello and I. W. Hamley, *Biomacromolecules*, 2024, submitted.
2. S. W. Provencher, *Comput. Phys. Commun.*, 1982, **27**, 229.
3. N. P. Cowieson, C. J. C. Edwards-Gayle, K. Inoue, N. S. Khunti, J. Doutch, E. Williams, S. Daniels, G. Preece, N. A. Krumpa, J. P. Sutter, M. D. Tully, N. J. Terrill and R. P. Rambo, *J. Synchrotron Rad.*, 2020, **27**, 1438-1446.
4. I. Bressler, J. Kohlbrecher and A. F. Thünemann, *J. Appl. Cryst.*, 2015, **48**, 1587-1598.
5. J. Kohlbrecher and I. Bressler, *J. Appl. Cryst.*, 2022, **55**, 1677-1688.
